# Antibiotic use and gut microbiome composition links from individual-level prescription data of 14,979 individuals

**DOI:** 10.1038/s41591-026-04284-y

**Published:** 2026-03-11

**Authors:** Gabriel Baldanzi, Anna Larsson, Sergi Sayols-Baixeras, Koen F. Dekkers, Ulf Hammar, Diem Nguyen, Tíscar Graells, Shafqat Ahmad, Camila Gazolla Volpiano, Guillaume Meric, Josef D. Järhult, Thomas Tängdén, Jonas F. Ludvigsson, Lars Lind, Johan Sundström, Karl Michaëlsson, Johan Ärnlöv, Beatrice Kennedy, Marju Orho-Melander, Tove Fall

**Affiliations:** 1 Molecular Epidemiology, Department of Medical Sciences, Uppsala University, Uppsala, Sweden; 2 Department of Clinical Sciences in Malmö, Lund University, Malmö, Sweden; 3 Medical Epidemiology, Department of Surgical Sciences, Uppsala University, Uppsala, Sweden; 4 CIBER Cardiovascular Diseases, Instituto de Salud Carlos III, Madrid, Spain; 5 Division of Family Medicine and Primary Care, Department of Neurobiology, Care Sciences and Society, Karolinska Institutet, Huddinge, Sweden; 6 Preventive Medicine Division, Harvard Medical School, Brigham and Women’s Hospital, Boston, MA USA; 7 School of Natural Sciences, Technology and Environmental Studies, Södertörn University, Huddinge, Sweden; 8 Cambridge Baker Systems Genomics Initiative, Baker Heart and Diabetes Institute, Melbourne, Victoria Australia; 9 Department of Cardiometabolic Health, University of Melbourne, Melbourne, Victoria Australia; 10 Department of Cardiovascular Research, Translation and Implementation, La Trobe University, Melbourne, Victoria Australia; 11 Department of Life Sciences, University of Bath, Claverton Down, Bath, UK; 12 Zoonosis Science Center, Department of Medical Sciences, Uppsala University, Uppsala, Sweden; 13 Infectious Diseases, Department of Medical Sciences, Uppsala University, Uppsala, Sweden; 14 Department of Medical Epidemiology and Biostatistics, Karolinska Institutet, Stockholm, Sweden; 15 Department of Pediatrics, Örebro University Hospital, Örebro, Sweden; 16 Division of Digestive and Liver Disease, Department of Medicine, Columbia University Medical Center, New York, NY USA; 17 Clinical Epidemiology, Department of Medical Sciences, Uppsala University, Uppsala, Sweden; 18 The George Institute for Global Health, University of New South Wales, Sydney, New South Wales Australia; 19 Center for Clinical Research Dalarna, Uppsala University, Falun, Sweden; 20 School of Health and Social Studies, Dalarna University, Falun, Sweden; 21 Science for Life Laboratory, Uppsala University, Uppsala, Sweden

**Keywords:** Epidemiology, Metagenomics, Antibiotics

## Abstract

Disruptions in gut microbiome are implicated in cardiometabolic disorders and other health outcomes. Antibiotics are known gut microbiome disruptors, but their long-term consequences remain underexplored. Here we combined individual-level data from the Swedish Prescribed Drug Register with fecal metagenomes of 14,979 adults to examine the association between oral antibiotic use over 8 years and gut microbiome. In multivariable confounder-adjusted regression models, antibiotic use <1 year before fecal sampling was associated with the greatest reduction in species diversity, but significant associations were also observed for use 1–4 and 4–8 years earlier. Clindamycin, fluoroquinolones and flucloxacillin accounted for most of the associations with the abundance of individual species. Use of these antibiotics 4–8 years earlier was associated with altered abundance of 10–15% of the species studied; penicillin V, extended-spectrum penicillins and nitrofurantoin were associated with only a few species. Similar results were found comparing one antibiotic course 4–8 years before sampling versus none in the past 8 years. These findings indicate that antibiotics may have long-lasting consequences for the gut microbiome.

## Abstract (web summary)

Using individual-level data from the Swedish Prescribed Drug Register and fecal metagenomes of 14,979 individuals in Sweden, the authors examined the association between oral antibiotic use over 8 years and gut microbiome and found evidence that antibiotics can have long-lasting impacts on the gut microbiome.

## Main

In observational studies, recurrent and long-term use of antibiotics has been associated with an increased risk of obesity^1,2^, type 2 diabetes^1,3,4^, cardiovascular disease^5^ and colorectal polyps and cancer^6,7^, potentially due to disruptions to the gut microbiome^8^. This hypothesis is supported by evidence linking the gut microbiome to human health, including obesity^9^, cardiometabolic disorders^10,11^, autoimmune conditions^12^ and colorectal cancer (CRC)^13,14^.

Smaller intervention studies in healthy volunteers have reported drastic alterations in the gut microbiome a few days after a course of oral antibiotics, particularly reductions in species diversity^15^ and microbial gene richness^16^. Other short-term alterations include increased abundance of potential pathogens such as *Escherichia coli*^17^; reduced abundance of the genera *Dialister*, *Veillonella* and *Eubacterium*^18^; enrichment of antimicrobial-resistance genes^15^; and increased risk of *Clostridium difficile* infection^19^.

Although the short-term antimicrobial effects of antibiotics are well-recognized, population-based investigations examining their long-term consequences on gut microbiome have not been conducted at scale^20^. Here, we assessed how oral antibiotic use in the 8 years before fecal sampling was associated with gut microbiome composition, while controlling for factors linked to high use of antibiotics, such as use of non-antibiotic medications and comorbidities. Information from the National Prescribed Drug Register (NPDR), which captures all antibiotics and other prescription medications dispensed to outpatients in Sweden^21^, was combined with gut microbiome data obtained by fecal deep shotgun metagenomics in three Swedish population-based cohorts. We found evidence linking antibiotic use 1–4 years and 4–8 years earlier to the gut microbiome composition at the time of fecal sampling. This link was observed even among individuals who had only a single antibiotic course 4–8 years earlier.

## Results

### Study population and antibiotic use

We studied the association between oral antibiotic use in the past 8 years and the gut microbiome composition using fecal metagenomics data that were collected as part of three population-based cohorts in Sweden: Swedish CArdioPulmonary bioImage Study^22^ (SCAPIS, *n* = 8,488), Swedish Infrastructure for Medical Population-based Life-course and Environmental Research^23^ (SIMPLER, *n* = 4,784) and the Malmö Offspring Study^24^ (MOS, *n* = 1,707). Descriptive characteristics for the 14,979 individuals are shown in Table 1. Information about recruitment, fecal sample collection and storage, DNA extraction and sequencing, as well as metagenomic profiling is described in Methods.

**Table 1:** Characteristics of participants at the time of fecal sampling

Characteristics	Number of antibiotic courses in past 8 years								
	SCAPIS			SIMPLER			MOS		
	0	1	≥2	0	1	≥2	0	1	≥2
Basic model (*n*)	2,573 (30.3%)	1,907 (22.5%)	4,008 (47.2%)	1,257 (26.3%)	1,073 (22.4%)	2,454 (51.3%)	478 (28.0%)	376 (22%)	853 (50%)
Age (years)	56.9 [53.4;61.1]	57.4 [53.5;61.1]	58.0 [54.0;61.6]	72.0 [70.0;75.0]	72.0 [70.0;75.0]	73.0 [71.0;75.0]	38.4 [26.9;51.4]	40.9 [27.8;52.6]	40.1 [27.9;52.4]
Female	1,100 (42.8%)	941 (49.3%)	2,376 (59.3%)	525 (41.8%)	487 (45.4%)	1,270 (51.8%)	184 (38.5%)	182 (48.4%)	530 (62.1%)
Smoker									
Never	1,464 (56.9%)	1,032 (54.1%)	1,904 (47.5%)	685 (54.5%)	543 (50.6%)	1,241 (50.6%)	333 (69.7%)	235 (62.5%)	484 (56.7%)
Former	804 (31.2%)	655 (34.3%)	1,546 (38.6%)	487 (38.7%)	445 (41.5%)	1,026 (41.8%)	84 (17.6%)	93 (24.7%)	229 (26.8%)
Current	305 (11.9%)	220 (11.5%)	558 (13.9%)	85 (6.8%)	85 (7.9%)	187 (7.6%)	61 (12.8%)	48 (12.8%)	140 (16.4%)
Highest education									
Compulsory	225 (8.7%)	175 (9.2%)	393 (9.8%)	533 (42.4%)	454 (42.3%)	900 (36.7%)	26 (5.4%)	16 (4.3%)	63 (7.4%)
Upper secondary	1,150 (44.7%)	826 (43.3%)	1,798 (44.9%)	376 (29.9%)	306 (28.5%)	760 (31.0%)	259 (54.2%)	224 (59.6%)	470 (55.1%)
University	1,198 (46.6%)	906 (47.5%)	1,817 (45.3%)	348 (27.7%)	313 (29.2%)	794 (32.4%)	193 (40.4%)	136 (36.2%)	320 (37.5%)
Born in Scandinavia^a^	2,153 (83.7%)	1,612 (84.5%)	3,337 (83.3%)	1,211 (96.3%)	1,034 (96.4%)	2,365 (96.4%)	473 (99.0%)	370 (98.4%)	843 (98.8%)
Full model (*n*)	2,573 (30.3%)	1,907 (22.5%)	4,008 (47.2%)	1,255 (26.2%)	1,073 (22.5%)	2,451 (51.3%)	478 (28.0%)	376 (22%)	853 (50%)
BMI (kg m^−^^2^)	26.3 [24.0;29.0]	26.5 [24.2;29.4]	26.8 [24.1;29.9]	25.6 [23.3;28.3]	25.8 [23.6;28.4]	26.3 [23.9;29.1]	24.7 [22.7;27.5]	25.2 [22.9;28.4]	25.0 [22.4;28.4]
Charlson Index ≥2	104 (4.0%)	120 (6.3%)	338 (8.4%)	104 (8.3%)	113 (10.5%)	462 (18.8%)	8 (1.7%)	4 (1.1%)	28 (3.3%)
Metformin	60 (2.3%)	61 (3.2%)	168 (4.2%)	64 (5.1%)	58 (5.4%)	161 (6.6%)	4 (0.8%)	4 (1.1%)	21 (2.5%)
Beta-blocker	178 (6.9%)	138 (7.2%)	419 (10.5%)	262 (20.9%)	254 (23.7%)	631 (25.7%)	16 (3.3%)	17 (4.5%)	37 (4.3%)
SSRI	131 (5.1%)	127 (6.7%)	391 (9.8%)	32 (2.5%)	44 (4.1%)	176 (7.2%)	18 (3.8%)	13 (3.5%)	48 (5.6%)
Statins	230 (8.9%)	192 (10.1%)	490 (12.2%)	360 (28.7%)	307 (28.6%)	788 (32.2%)	21 (4.4%)	17 (4.5%)	41 (4.8%)
Antipsychotics	20 (0.8%)	12 (0.6%)	45 (1.1%)	5 (0.4%)	4 (0.4%)	16 (0.7%)	4 (0.8%)	0 (0.0%)	3 (0.4%)
PPI	149 (5.8%)	188 (9.9%)	653 (16.3%)	121 (9.6%)	145 (13.5%)	541 (22.1%)	29 (6.1%)	26 (6.9%)	95 (11.1%)
Polypharmacy^b^	97 (3.8%)	89 (4.7%)	421 (10.5%)	78 (6.2%)	97 (9.0%)	343 (14.0%)	8 (1.7%)	8 (2.1%)	38 (4.5%)

Continuous variables are presented as median [25th–75th percentile], and categorical variables are presented as *n* (%). SCAPIS, Swedish CArdioPulmonary bioImage Study; SIMPLER, Swedish Infrastructure for Medical Population-based Life-course Environmental Research; MOS, Malmö Offspring Study; BMI, body mass index; SSRI, selective serotonin reuptake inhibitor; PPI, proton-pump inhibitor use in the past year. ^a^In MOS, ‘born in Sweden’ was used instead. ^b^Use of ≥5 medications.

**Extended Data Fig. 1:**
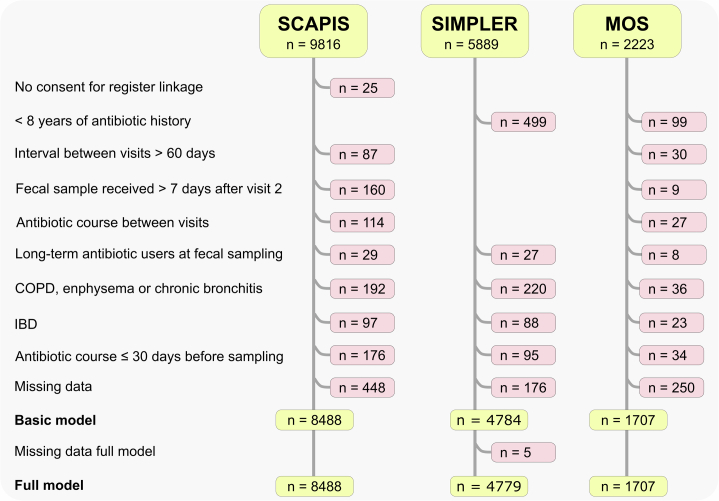
Flowchart of exclusion of participants. Flowchart of exclusion of participants and final sample size for the basic and full models. Yellow boxes show the number of participants in the studies and those who were included in the analysis with the basic or full model. Red boxes show the number of participants excluded in each step. COPD: chronic pulmonary obstructive disease, IBD: inflammatory bowel disease, MOS: Malmö Offspring Study, SCAPIS: Swedish CArdioPulmonary bioImage Study, SIMPLER: Swedish Infrastructure for Medical Population-based Life-course and Environmental Research.

To exclude individuals using antibiotics at the time of sampling or with an ongoing infection, we excluded participants who had dispensed prescriptions of antibiotics in the 30 days before the visit to the test center. Participants with inflammatory bowel disease (IBD) and chronic pulmonary disease were also excluded. For the full list of exclusion criteria and number of exclusions, see Methods and Extended Data Fig. 1. Antibiotic use data were provided by the NPDR, which includes all oral antibiotics dispensed to outpatients in Sweden since 2005. Because recruitment in SCAPIS and MOS started in 2013, we limited the history of antibiotic use to the 8 years before the visit to the test center. The most-prescribed antibiotics to this study population were penicillin V, extended-spectrum penicillins and tetracyclines (Table 2). During the study period, the number of dispensed antibiotic prescriptions declined over time in the study population, except nitrofurantoin, which increased (Supplementary Fig. 1). The proportion of participants who had used any antibiotic at least once in the past 8 years ranged between 69.7% in SCAPIS and 73.7% in SIMPLER (Table 1).

**Table 2:** Antibiotic use in the population-based cohorts by period before fecal sampling

	SCAPIS			SIMPLER			MOS		
Period	<1 year	1–4 years	4–8 years	<1 year	1–4 years	4–8 years	<1 year	1–4 years	4–8 years
Penicillin V	559 (6.6%)	1,501 (17.7%)	2,124 (25%)	272 (5.7%)	915 (19.1%)	1,266 (26.5%)	109 (6.4%)	332 (19.4%)	482 (28.2%)
Tetracyclines	287 (3.4%)	858 (10.1%)	1,405 (16.6%)	146 (3.1%)	420 (8.8%)	704 (14.7%)	60 (3.5%)	189 (11.1%)	265 (15.5%)
Penicillins ES	301 (3.5%)	824 (9.7%)	1,013 (11.9%)	201 (4.2%)	559 (11.7%)	615 (12.9%)	48 (2.8%)	149 (8.7%)	209 (12.2%)
Flucloxacillin	166 (2%)	570 (6.7%)	732 (8.6%)	120 (2.5%)	349 (7.3%)	428 (8.9%)	28 (1.6%)	104 (6.1%)	142 (8.3%)
Fluoroquinolones	136 (1.6%)	384 (4.5%)	506 (6%)	126 (2.6%)	361 (7.5%)	480 (10%)	12 (0.7%)	56 (3.3%)	69 (4%)
Nitrofurantoin	138 (1.6%)	374 (4.4%)	331 (3.9%)	113 (2.4%)	288 (6%)	272 (5.7%)	23 (1.3%)	87 (5.1%)	61 (3.6%)
Clindamycin	104 (1.2%)	298 (3.5%)	387 (4.6%)	37 (0.8%)	131 (2.7%)	160 (3.3%)	20 (1.2%)	64 (3.7%)	83 (4.9%)
Sulfamethoxazole-trimethoprim	38 (0.4%)	142 (1.7%)	209 (2.5%)	46 (1%)	132 (2.8%)	196 (4.1%)	2 (0.1%)	27 (1.6%)	52 (3%)
Cephalosporins	46 (0.5%)	142 (1.7%)	247 (2.9%)	19 (0.4%)	52 (1.1%)	119 (2.5%)	16 (0.9%)	45 (2.6%)	77 (4.5%)
Macrolides	42 (0.5%)	132 (1.6%)	243 (2.9%)	12 (0.3%)	51 (1.1%)	81 (1.7%)	15 (0.9%)	42 (2.5%)	65 (3.8%)
Amoxicillin-clavulanic acid	33 (0.4%)	76 (0.9%)	111 (1.3%)	9 (0.2%)	39 (0.8%)	60 (1.3%)	2 (0.1%)	9 (0.5%)	17 (1%)

Number of individuals who had at least one antibiotic course in the 8 years before fecal sampling in the three cohorts, in each time period. <1 year: <1 year before fecal sampling (1-year period); 1–4 years: ≥1 and <4 years before fecal sampling (3-year period); 4–8 years: ≥4 and <8 years before fecal sampling (4-year period). Penicillins ES, extended-spectrum penicillins.

### Recent and past use of antibiotics were associated with a lower diversity of gut microbiome species

To decide which covariates to include in our statistical models, we created two directed acyclic graphs (DAGs)^25^. Because the covariates were primarily assessed at the time of fecal sampling, the basic model DAG (Supplementary Fig. 2a) focused on temporally stable covariates, whereas the full-model DAG (Supplementary Fig. 2b) also accounted for the potential confounding of comorbidities and medications^26^. Based on the DAG, the basic model included age, sex, education, smoking and country of birth. Test-site-specific analysis plates were included in the models to account for batch effects within each cohort, because this was the technical covariate explaining the largest proportion of microbiome variation. The full model additionally included body mass index (BMI), Charlson Comorbidity Index^27,28^, polypharmacy^26,29,30^ and use of the following medications: proton-pump inhibitors (PPIs)^31^, metformin, selective serotonin reuptake inhibitors (SSRIs), statins, beta-blockers and antipsychotics^26,29,30,32–34^. These are commonly used medications, except for antipsychotics, and have been shown by us and others to be associated with the gut microbiome composition^26,29,30,32–34^. Antipsychotic medication was added because it has been linked to gut microbiome alterations and a potential increased risk of bacterial infections^35^. Polypharmacy was defined as the use of ≥5 different medications at the time of fecal sampling. This variable was previously associated with the gut microbiome composition^29,30^ and is a marker of multimorbidity. To account for several comorbidities, we calculated the Charlson Comorbidity Index primarily based on patient register data. This index captures conditions such as diabetes, cancer, cardiovascular disease, liver disease and renal disease^28^. Use of non-antibiotic medications was either self-reported or retrieved from the NPDR based on prescriptions dispensed within 1 year before fecal sampling.

**Fig. 1:**
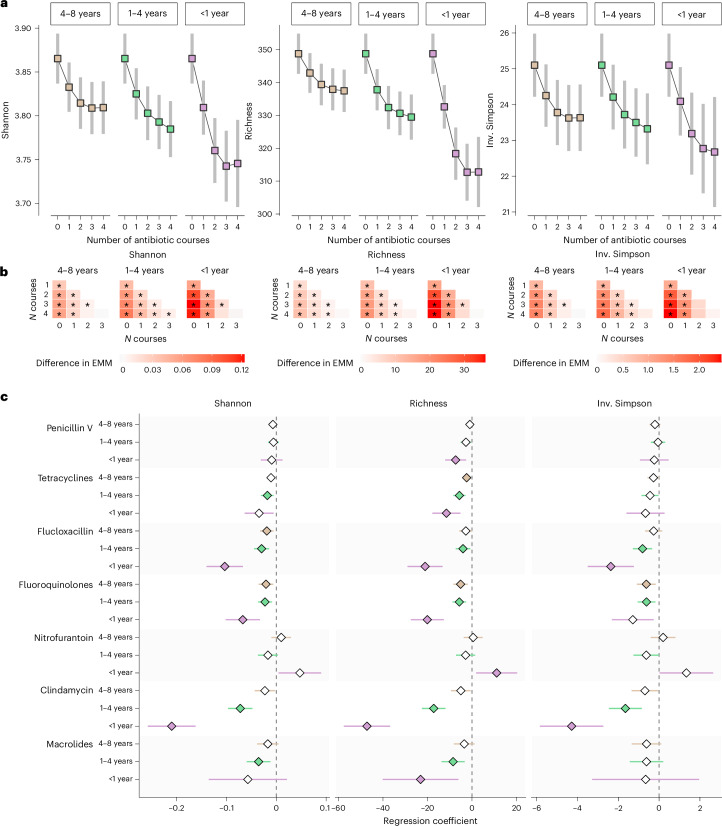
Antibiotic use and gut microbiome species diversity. **a**, Microbiome diversity metrics (Shannon Index, species richness and inverse (Inv.) Simpson Index) for each additional course of any antibiotic 4–8 years, 1–4 years and <1 year before fecal sampling. Estimated marginal means (EMMs) of diversity were obtained using regression models, with antibiotic exposure modeled using restricted cubic splines and adjustment for age, sex, smoking, education, country of birth, site-specific analysis plate, BMI, Charlson Comorbidity Index, polypharmacy and use of PPIs, metformin, SSRIs, statins, beta-blockers and antipsychotics (*n* = 14,974). Squares represent the EMMs, and bars the 95% confidence intervals. **b**, Pairwise differences in EMMs of microbiome diversity by number of previous antibiotic courses. Stars indicate significant differences (FDR < 5%). **c**, Associations between antibiotic use in the 8 years before fecal sampling and gut microbiome species diversity were investigated using regression models adjusted for the same covariates as above, in three cohorts (SCAPIS, SIMPLER, MOS, total *n* = 14,974), followed by meta-analyses of the regression coefficients. The *x* axis and diamonds display the meta-analyzed regression coefficients; error bars represent 95% confidence intervals. Filled symbols indicate statistically significant associations (FDR < 5%). Antibiotics with at least one such association are shown.

We first estimated (marginal means) the diversity of species in the gut microbiome (alpha diversity) for each additional antibiotic course within three periods: <1 year, ≥1 and <4 years (1–4 years) and ≥4 and <8 years (4–8 years) before fecal sampling. Antibiotic exposure was modeled as three continuous variables representing the number of prescriptions within each period, and the variables were included in the same linear regression using restricted cubic splines and adjusted for covariates (full model). We observed that the estimated diversity of gut microbiome species decreased with each additional antibiotic course within the three periods. The estimated decrease was greater for the first two courses than for the third and fourth courses (Fig. 1a,b).

As the effect of oral antibiotics on the gut microbiome varies by mechanism of action and pharmacokinetics, we continued our analysis by dividing the antibiotics into 11 classes. Correlation plots for the number of prescriptions by antibiotic class are provided in Supplementary Figs. 3–5. Analyses were performed separately for each cohort, and regression coefficients were combined using inverse-variance weighted fixed-effect meta-analysis. Regression coefficients were consistent between basic and full models (Spearman correlation = 0.95; Supplementary Table 1) and across cohorts (Supplementary Fig. 6). Unless otherwise stated, results refer to the full model. We found that the use of 6 of the 11 antibiotic classes <1 year before fecal sampling was associated with a lower species diversity in at least one diversity metric, after multiple testing correction considering a false discovery rate (FDR) < 5% (Fig. 1c and Supplementary Table 1). Lower diversity of gut microbiome species has been associated with a range of health conditions, such as obesity, diabetes and IBD^36–38^. Clindamycin, fluoroquinolones and flucloxacillin had the largest effect estimates. In the results for species richness, each course of clindamycin <1 year before fecal sampling was associated with an average of 47 fewer species detected (*q-*value = 2.1 × 10^−17^; Fig. 1c). Each course of fluoroquinolones or flucloxacillin was associated with an average of 20 and 21 fewer species detected (*q-*value = 1.3 × 10^−6^ and 1.4 × 10^−6^, respectively). Fluoroquinolones, flucloxacillin and tetracyclines use 1–4 years and 4–8 years before fecal sampling were also associated with lower diversity (Fig. 1c), as were clindamycin and macrolide use 1–4 years, but not 4–8 years, before fecal sampling. Positive associations with species diversity were observed for nitrofurantoin use <1 year before fecal sampling (*q*-value = 0.043). However, given the lack of confirmation in subsequent analysis in this study, such as the single antibiotic course use, this association should be interpreted with caution, and we hypothesize that this association is likely due to chance or a possible collider bias. No associations were detected for extended-spectrum penicillins (that is, pivmecillinam and amoxicillin), amoxicillin-clavulanic acid or sulfamethoxazole-trimethoprim (Supplementary Table 1). Amoxicillin-clavulanic acid is a broad-spectrum antibiotic that has marked short-term effects on the gut microbiome^39^. The lack of association in the current study could be due to its infrequent use in outpatients in Sweden compared to other countries^40^.

Results similar to the main analysis were observed in sex- and age-stratified analyses. There was some evidence for a stronger negative association in women with species diversity for amoxicillin-clavulanic acid use <1 year, fluoroquinolones 1–4 years and flucloxacillin 4–8 years before fecal sampling (interaction *P* value = 0.01, likelihood-ratio test *q*-value = 0.08; Supplementary Fig. 7 and Supplementary Tables 2 and 3).

To assess the impact of alternative exclusion criteria based on recent antibiotic use, we applied four thresholds: no exclusion, and exclusion of individuals who had used antibiotics within 30 days (*n* = 284), 6 months (*n* = 1,605) or 12 months (*n* = 2,908) before fecal sampling. The effect estimates for exposures occurring 4–8 years and 1–4 years before fecal sampling remained largely unchanged. For clindamycin use <1 year before sampling, exclusion of the most recent antibiotic users attenuated the regression coefficients (Supplementary Fig. 8 and Supplementary Table 4). No clear attenuation was observed for other antibiotics, although low precision of the estimates limited interpretability in some cases.

**Extended Data Fig. 2:**
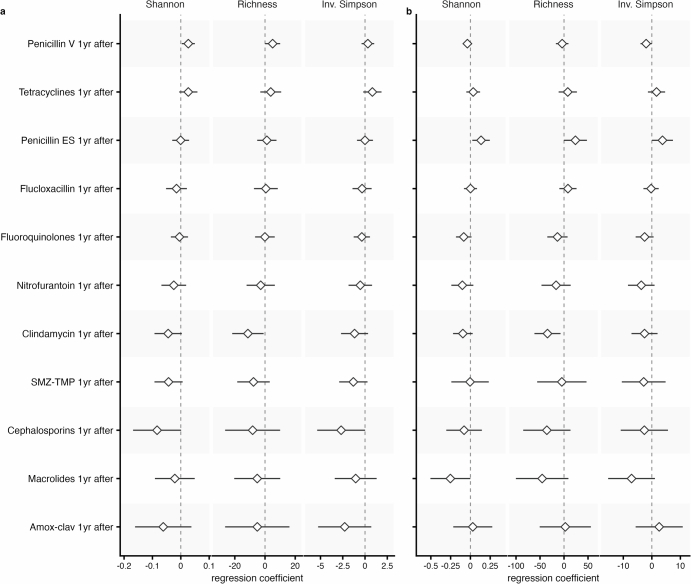
Negative control exposures. Associations between antibiotic use in the 1 year after fecal sampling and gut microbiome species diversity (Shannon index, species richness, and inverse Simpson index). Analyses were performed separately by cohort, and regression coefficients were meta-analyzed using fixed-effects models. None of the associations were significant after multiple testing adjustment (unfilled shapes, FDR > 5%). The x-axis and diamonds display the meta-analyzed regression coefficients; error bars represent 95% confidence intervals. **a**. Associations investigated using regression models adjusted for antibiotic use in periods <1 year, 1–4 years, and 4–8 years before fecal sampling, age, sex, smoking, education, country of birth, site-specific analysis plate, BMI, Charlson Comorbidity Index, polypharmacy, and use of proton-pump inhibitors, metformin, selective serotonin reuptake inhibitors, statins, beta-blockers, and antipsychotics (n = 13,511). **b**. Associations after excluding all participants with an antibiotic prescription before fecal sampling (n included= 3,803). Amox-clav: amoxicillin-clavulanic acid. Penicillin ES: extended-spectrum penicillins; SMZ-TMP: sulfamethoxazole-trimethoprim.

To examine whether the covariate adjustment fully controlled for differences between individuals exposed and unexposed to antibiotics, antibiotic use within 1 year after fecal sampling was used as a negative control exposure. Antibiotic use after sampling was not associated with species diversity, indicating that the full model likely controlled for the most important confounding effects (Extended Data Fig. 2 and Supplementary Tables 5 and 6). No multicollinearity issues were detected using the generalized variance inflation factor (Supplementary Table 1). In the sensitivity analyses, removing 540 individuals who were hospitalized for infection or 5,132 hospitalized for any reason in the past 8 years, the overall effect estimates were similar (Supplementary Table 7 and Supplementary Fig. 9).

### Association between antibiotic use and gut microbiome diversity over time using a functional regression model

**Fig. 2:**
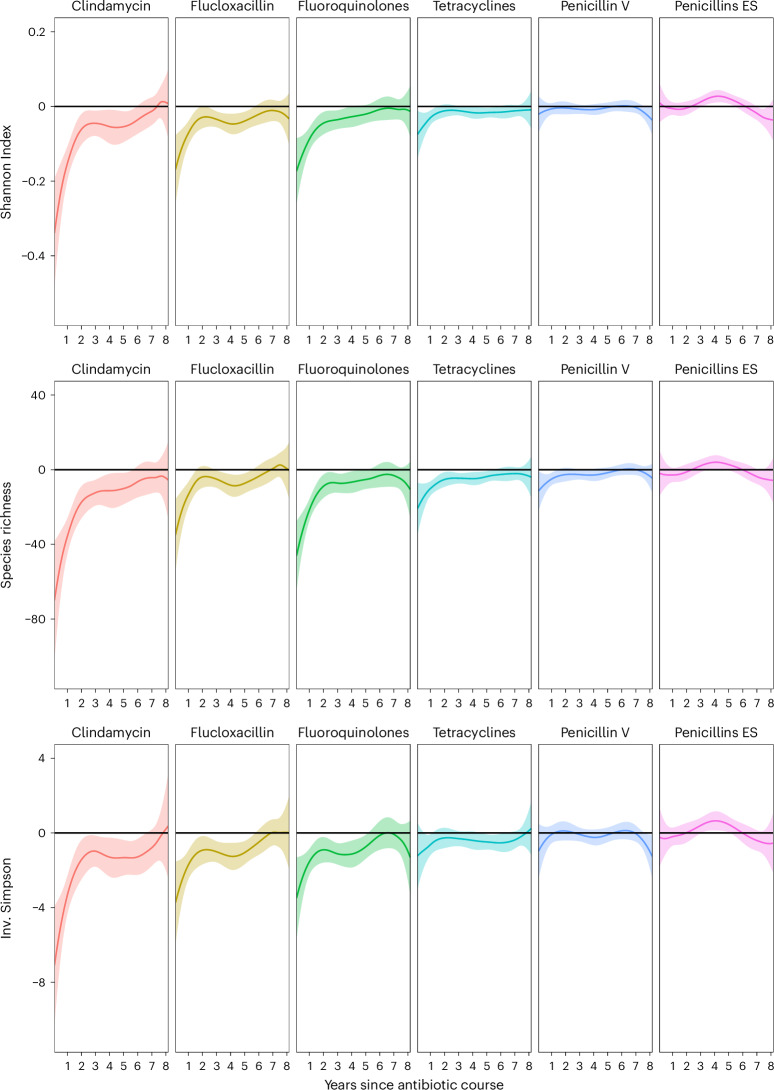
Association between antibiotic use and gut microbiome diversity over time. Functional regression model examining the association between antibiotic classes and gut microbiome diversity (Shannon index, species richness and inverse Simpson index) since the antibiotic course adjusted for full-model covariates in three cohorts (SCAPIS, SIMPLER, MOS, total *n* = 14,974), followed by meta-analyses of the regression coefficients. The *y* axis and solid lines represent the meta-analyzed functional regression coefficients for antibiotic exposure at each time point; the shaded area represents the 95% confidence interval band.

To explore the recovery of microbiome diversity after antibiotic use, we implemented a functional regression model leveraging the high correlation between temporally adjacent regression coefficients by fitting a cubic spline. Antibiotic classes that were infrequently prescribed—namely, cephalosporins, macrolides, amoxicillin-clavulanic acid, sulfamethoxazole-trimethoprim and nitrofurantoin—were merged into a single predictor variable to ensure model stability. The results indicated that gut microbiome diversity recovered most rapidly within the first 2 years following antibiotic exposure, with a markedly slower recovery observed in subsequent years. This pattern was evident for richness after clindamycin, fluoroquinolones and tetracycline use. Moreover, the analysis suggested that the recovery rate in the period following the antibiotic use was proportional to the magnitude of the initial reduction in diversity (Fig. 2), consistent with another study showing that the magnitude of microbiome disruption immediately after antibiotic treatment predicted the duration of its effect^41^.

### A single course of antibiotics in the past 8 years was associated with lower species diversity in the gut

**Extended Data Fig. 3:**
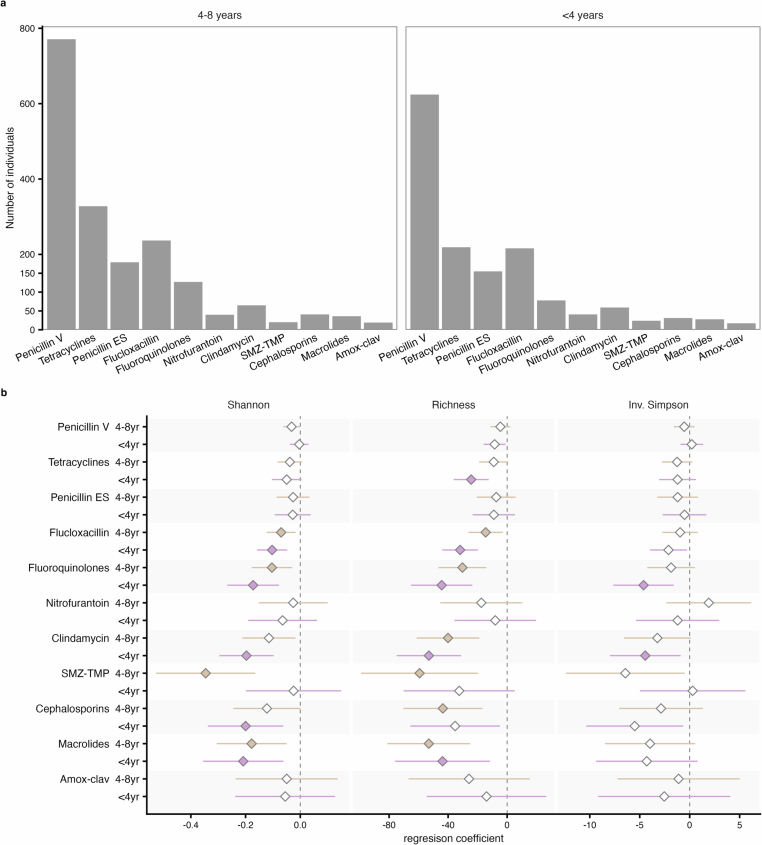
Single antibiotic course and its associations with gut microbiome species diversity. **a**. Number of individuals exposed to a single antibiotic course <4 and 4-8 years before fecal sampling. **b**. Associations between antibiotic use and gut microbiome species diversity (Shannon index, species richness, and inverse Simpson index) in participants who had 1 or no antibiotic course in the 8 years before fecal sampling in the three cohorts (SCAPIS, MOS, and SIMPLER, total n = 7,664). Associations were investigated using regression models adjusted for age, sex, smoking, education, country of birth, site-specific analysis plate, BMI, Charlson Comorbidity Index, polypharmacy, and use of proton-pump inhibitors, metformin, selective serotonin reuptake inhibitors, statins, beta blockers, and antipsychotics, followed by meta-analyses of the regression coefficients. The x-axis and the diamonds display the meta-analyzed regression coefficients; error bars represent 95% confidence intervals. Filled shapes indicate significant associations (FDR < 5%). Amox-clav: amoxicillin-clavulanic acid. Penicillin ES: extended-spectrum penicillins; SMZ-TMP: sulfamethoxazole-trimethoprim.

In an additional analysis, we restricted the sample to those 7,664 participants who had only one antibiotic course (*n* = 3,356) or none (*n* = 4,308) in the 8 years before fecal sampling, as this was a more homogenous population (Table 1). To ensure statistical power for antibiotic classes that are less often prescribed, the periods 1–4 and <1 year were merged. A single course of tetracyclines, flucloxacillin, fluoroquinolones, clindamycin, sulfamethoxazole-trimethoprim, cephalosporins or macrolides <4 years or 4–8 years before sampling was associated with lower microbiome species diversity (Extended Data Fig. 3 and Supplementary Table 8). Although the antimicrobial spectrum is considerably different between the cephalosporin generations, the low number of individuals (*n* = 72) exposed to a single course of cephalosporins in the past 8 years hindered analyses into the generations separately.

### Clindamycin, flucloxacillin and fluoroquinolones had the highest number of associations with the abundance of gut microbiome species

**Fig. 3:**
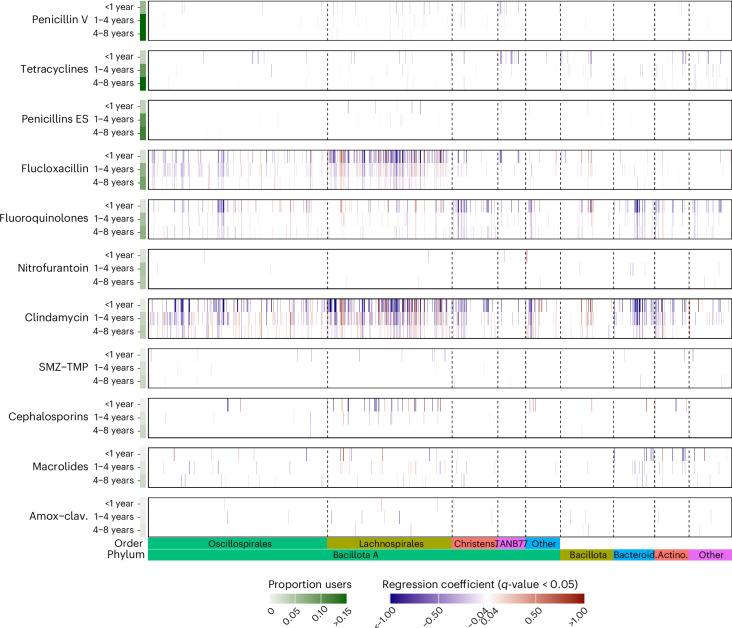
Antibiotic use and its associations with abundance of gut microbiome species. The associations between the number of antibiotic courses before fecal sampling and species abundance were investigated using regression models adjusted for full-model covariates in three cohorts (SCAPIS, SIMPLER and MOS, total *n* = 14,974), followed by meta-analyses of the regression coefficients. Blue or red vertical bars represent a negative or positive meta-analyzed regression coefficient, respectively. Only significant associations are shown (FDR < 5%). The *y* axis displays the 11 antibiotic classes and the periods of the antibiotic courses: <1 year, 1–4 years and 4–8 years before fecal sampling. The proportion of users indicates the proportion of participants from the study population who had at least one course of the respective antibiotic in that period. The 1,340 species in the *x* axis were ordered based on their taxonomy. Phyla are identified at the bottom of the plot. For the phylum Bacillota A, the taxonomic orders are also displayed. SMZ-TMP, sulphamethoxazole-trimethoprim; Amox-clav., amoxicillin-clavulanic acid; Bacteroid., Bacteroidota; Actino., Actinomycetota; Christens., Christensenellales.

We next investigated the associations between exposure to the 11 antibiotic classes and the abundance of 1,340 species present in >2% of the participants in the three cohorts. Regression coefficients of the basic and full models were highly consistent (Spearman correlation = 0.99); hence, we refer to the results of the full model. Clindamycin, flucloxacillin and fluoroquinolones accounted for most of the associations between antibiotic use and individual species abundances: that is, 37.9%, 25.8% and 17.9% of the FDR < 5% associations, respectively (Fig. 3 and Supplementary Table 9). Although associations for antibiotic use <1 year before fecal sampling were the strongest in terms of effect estimates and *P* values, many associations were also observed for antibiotic use 1–4 and 4–8 years before sampling (Fig. 3 and Supplementary Table 9). Clindamycin use <1 year before sampling was associated with 296 of the 1,340 species analyzed, flucloxacillin with 203 species and fluoroquinolones with 172 species. For comparison, penicillin V, the most-prescribed antibiotic, was associated with only 29 species. Most associations were in the negative direction (that is, decreased relative abundance of the species), but positive associations were also observed. For example, clindamycin use 1–4 years before sampling was associated with reduced relative abundance of 208 and increased relative abundance of 141 species.

Flucloxacillin is a beta-lactamase-resistant penicillin with a narrow spectrum targeting Gram-positive bacteria. This antibiotic was primarily associated with differences in the abundance of bacteria in the phylum Bacillota A, particularly in the orders Lachnospirales and Oscillopirales, which are predominantly Gram-positive (Fig. 3). In contrast, clindamycin and especially fluoroquinolones were associated with bacteria from a more diverse range of phyla, including many species in the phyla Bacteroidota and Actinomycetota (Fig. 3). Both fluoroquinolones and clindamycin are associated with a higher risk of *C. difficile* infection^19^. The strong associations with clindamycin use could be explained by the predominantly biliary excretion and the effect against anaerobes^42^. Fluoroquinolones are broad-spectrum antibiotics^43^ and their large impact on gut microbiome has been highlighted^36^.

Exclusion of individuals who had been hospitalized for infection produced nearly unchanged regression coefficients for the antibiotic–species associations. The Spearman correlation between sensitivity analysis and the full-model coefficients was ≥0.87 for all antibiotics (Supplementary Fig. 10a and Supplementary Table 10). Exclusion of individuals hospitalized for any reason also produced highly correlated coefficients between the sensitivity analysis and full-model coefficients for all antibiotics except amoxicillin-clavulanic acid and sulfamethoxazole-trimethoprim (Spearman correlation = 0.78 and 0.58, respectively) (Supplementary Fig. 10b and Supplementary Table 10).

**Extended Data Fig. 4:**
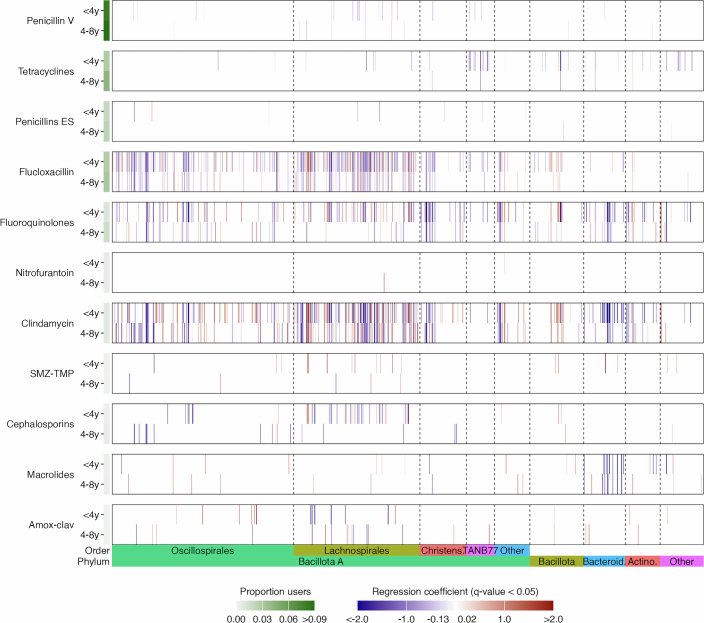
Single antibiotic course and its associations with gut microbiome species abundance. The associations between antibiotic use and species abundance in participants who had 1 or no antibiotic course in the 8 years before fecal sampling were investigated using regression models adjusted for full-model covariates in three cohorts (SCAPIS, MOS, and SIMPLER, total n = 7,664), followed by meta-analyses of the regression coefficients. Blue or red vertical bar represents a negative or positive meta-analyzed regression coefficient, respectively. Only significant associations are shown (FDR < 0.5). The y-axis displays the 11 antibiotic classes and the periods of the antibiotic courses: <4 years, and 4–8 years before fecal sampling. The proportion of users indicates the proportion of individuals who had a course of the respective antibiotic in that period among those who had 1 antibiotic course or none in the 8 years before sampling. The 1340 species in the x-axis were ordered based on their taxonomy. Phyla are identified at the bottom of the plot. For the phylum *Bacillota* A, the taxonomic orders are also displayed. Amox-clav: amoxicillin-clavulanic acid; Penicillins ES: extended-spectrum penicillins; SMZ-TMP: sulphamethoxazole-trimethoprim; Bacteroid.: *Bacteroidota*; Actino.: *Actinomycetota*; Christens: *Christensenellales*.

In the analysis restricted to individuals who had only one or no antibiotic course in the previous 8 years, clindamycin, flucloxacillin and fluoroquinolones were again the antibiotics with the largest number of species associations (Extended Data Fig. 4 and Supplementary Table 11). A single course of clindamycin, flucloxacillin or fluoroquinolones <4 years before sampling was associated with 256, 283 and 170 species, respectively. A single course of these antibiotics 4–8 years before sampling was associated with 196, 148 and 80 species, respectively.

### Age and sex differences in antibiotic–species associations

To explore potential age and sex differences in antibiotic–species associations, we conducted regression models and meta-analyses stratified by age (≤55 years or >55 years) and sex. Models included all full-model covariates, except sex in sex-stratified analyses. Furthermore, we tested for interactions between antibiotic use and both sex and age.

Overall, the pattern of associations was consistent across age and sex strata, with clindamycin, flucloxacillin and fluoroquinolones accounting for most associations (Supplementary Figs. 11 and 12 and Supplementary Tables 12 and 13). Evidence of interaction between antibiotic use and sex was detected for 74 species (*q*-value < 0.05 in the likelihood-ratio test comparing models with or without interaction terms; Supplementary Table 12). Most significant interactions indicated stronger associations in women, including 22 with clindamycin use, 15 with fluoroquinolones and 11 with flucloxacillin. Stronger associations in men were observed in nine clindamycin–sex interactions. (Supplementary Table 12).

For interactions between antibiotic use and age, we identified evidence of interactions in associations with 97 species. Clindamycin use accounted for many interactions, with 31 showing stronger associations in younger individuals and 22 in older individuals. Significant interactions indicating stronger associations in younger individuals were also observed in 20 fluoroquinolone–age and 7 flucloxacillin–age interactions. (Supplementary Table 13).

### Links between antibiotic-associated species and host health

Antibiotic use has been associated with a higher risk of diabetes and cardiovascular disease^3,5^. In the present study, use of clindamycin, fluoroquinolones and flucloxacillin was associated with a greater abundance of *Enterocloster bolteae*, *E. citroniae* (previously *Clostridium bolteae* and *C. citroniae*), *Flavonifractor plautii*, R*uminococcus* B *gnavus* and *Eggerthella lenta*. These species have been associated with a higher BMI, serum triglycerides (TG) and risk of type 2 diabetes^44–46^, although evidence of causality is lacking. Our results align with previous hypotheses that antibiotic-induced alterations in gut microbiome may contribute to the development of cardiometabolic diseases^1,3–5^.

**Fig. 4:**
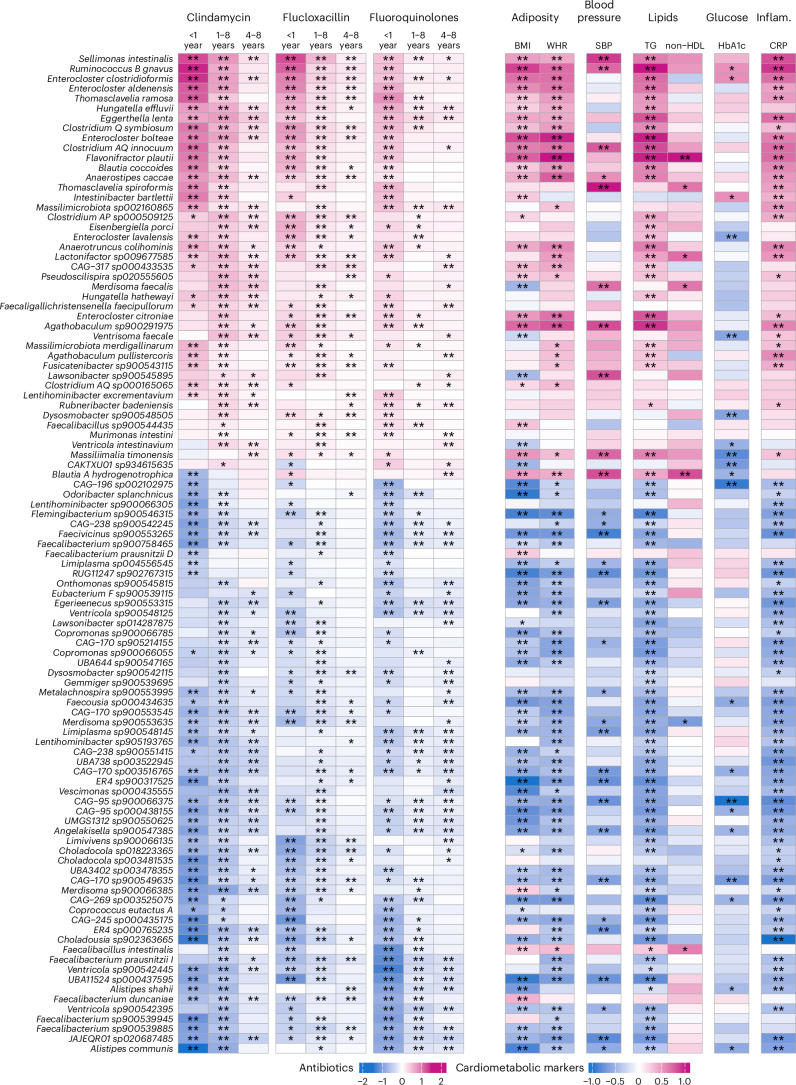
Association between the 101 species jointly associated with clindamycin, flucloxacillin and fluoroquinolones and cardiometabolic markers in the SCAPIS cohort. The heatmap shows the regression coefficients between antibiotic use and species and the partial Spearman correlation coefficients between species and cardiometabolic markers. Species in the *y* axis are organized by hierarchical clustering of the antibiotic–species associations. Stars denote significant associations (one star = FDR < 5%, two stars = FDR < 1%). Antibiotic–species associations were adjusted for full-model covariates; associations with cardiometabolic markers were adjusted for age, sex, BMI (except for the correlation with BMI), smoking, country of birth, education and site-specific analysis plate. SBP, systolic blood pressure; non-HDL, non-high-density lipoprotein cholesterol; HbA1c, glycated hemoglobin; Inflam., Inflammation.

To examine the link between the species associated with antibiotic use and cardiometabolic markers in SCAPIS, we focused on the 101 species associated with all three antibiotics at any period. Among species positively associated with antibiotic use, *Sellimonas intestinalis*, *R. B gnavus*, *E. clostridioformis*, *E. aldenensis* and *Thomasclavelia ramosa* were linked to higher BMI, waist–hip ratio (WHR), serum TG levels and C-reactive protein (CRP) levels (Fig. 4 and Supplementary Table 14). Overall, species negatively associated with antibiotic use were associated with lower BMI, WHR, TG and CRP levels. Among these species were *Alistipes communis* and *Odoribacter splanchnicus*.

**Extended Data Fig. 5:**
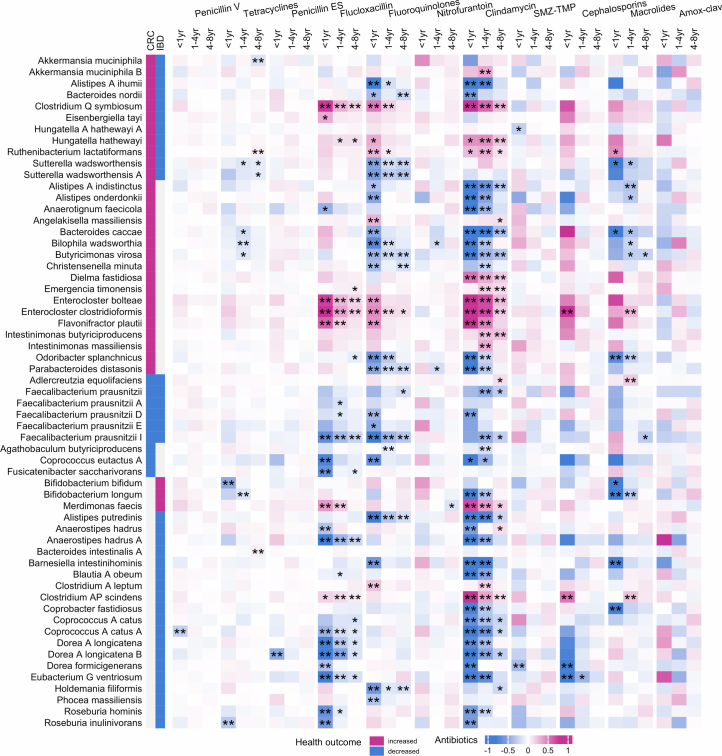
Antibiotic–species associations for bacterial species previously linked to colorectal cancer (CRC) or inflammatory bowel disease (IBD). Heatmap showing regression coefficients for associations between antibiotic use before fecal sampling and bacterial species previously linked to CRC^14^ or IBD^48^. ‘Increased’ or ‘decreased’ indicate the direction of association with the health outcome in models adjusted for age, sex, and BMI; gray boxes indicate no association. When a species linked to CRC or IBD matched multiple subspecies in our data, all corresponding subspecies are shown. The x-axis indicates the period when the antibiotic was used in relation to fecal sampling. Stars denote significant associations (one star = FDR < 5%; two stars = FDR < 1%). Antibiotic–species associations were adjusted for full-model covariates. Amox-clav: amoxicillin-clavulanic acid; Penicillins ES: extended-spectrum penicillins; SMZ-TMP: sulphamethoxazole-trimethoprim.

Besides cardiometabolic health, antibiotic use has been associated with an increased risk of CRC^7^ and IBD^7,47^, which are conditions linked to gut microbiome disruptions^14,48^. We leveraged our antibiotic–species association results to examine how antibiotic use relates to species previously linked to these conditions^14,48^. Species enriched in CRC showed both positive and negative associations with antibiotic use (Extended Data Fig. 5). *Fusobacterium nucleatum*, one of the most important signature species of CRC^14^, was not investigated in the current study because its prevalence was below 2% in the study population. For IBD, we observed a more consistent pattern where species depleted in IBD were negatively associated with antibiotic use, especially clindamycin. These findings align with previous evidence of an association between antibiotic use and increased risk of IBD^47^.

## Discussion

In this population-based study of 14,979 participants, we investigated the association between antibiotic use in the 8 years before fecal sampling and the composition of the gut microbiome. Three findings stand out. First, although the strongest associations were found for antibiotics used <1 year before sampling, antibiotics used 1–4 years and 4–8 years before sampling were also associated with lower diversity and differences in the abundance of species. Second, the associations were mainly related to three antibiotic classes: clindamycin, flucloxacillin and fluoroquinolones. Third, a single course of antibiotics 4–8 years before sampling was associated with the gut microbiome diversity and the abundance of certain species. These findings support the notion that the effect of antibiotics on the gut microbiome may persist for several years.

Microbiome resilience, how well the microbiome recovers after antibiotics, is not fully understood. A partial recovery often occurs within weeks^15,20,41,49^, but a full recovery might take years^41,49–51^. In intervention studies (*n* = 6–66), the duration of the reduction in the species diversity varied by antibiotic class^15,17,49,52^. In one study, diversity remained reduced for 4 months after clindamycin use and 12 months after fluoroquinolone use compared to pre-antibiotic levels, but no effect was observed after amoxicillin, an extended-spectrum penicillin^53^. Similarly, clindamycin and fluoroquinolone use were associated with lower gut microbiome species diversity in the current study, whereas the use of extended-spectrum penicillins was not. Interindividual variation in gut microbiome responses to antibiotics has been observed. In a study of cohabiting adults, the fluoroquinolone ciprofloxacin caused transient changes with recovery within a week for most participants, but about a quarter showed long-lasting effects, including colonization by external strains up to 2.5 years after exposure^41^.

Observational studies have described associations between antibiotic use over multiple years and gut microbiome. In the MetaCardis consortium (*n* = 2,173), antibiotic use in the past 5 years was ranked third, after diet and country of residence, in explaining variability in gut microbiome taxonomy^26^. In the Estonian Microbiome Cohort (*n* = 2,509), the number of antibiotic courses in the last 10 years was associated with lower gut microbiome species diversity^33^. However, neither of these studies differentiated recent antibiotic use from past use or performed analysis by antibiotic class. In our study, both recent use and antibiotic use 4–8 years before sampling were associated with the gut microbiome, and the associations differed by antibiotic classes. Additionally, we observed differences in antibiotic–species associations between sexes and age groups. These differences may reflect variations in baseline gut microbiome or differences in antibiotic pharmacokinetics across sexes and age, especially in older individuals^54^.

Differences in gut microbiome associations across antibiotic classes may be explained by differences in their spectrum of activity and pharmacokinetics. The strong associations for flucloxacillin are intriguing. Flucloxacillin is a narrow-spectrum penicillin characterized by a distinctive side chain that confers resistance to many beta-lactamases. As a result, its antibacterial activity is unique and may differ from other narrow-spectrum antibiotics, with regard to both pathogenic bacteria (for example, *Staphylococcus aureus* and anaerobic bacteria) and species that are not detectable by phenotypic methods. Another conceivable explanation for the possible pronounced impact of flucloxacillin is its variable bioavailability, ranging between 50% and 70% (ref. ^55^) and partial bile excretion, which theoretically can result in high fecal drug concentrations. However, data on intra-intestinal concentrations of flucloxacillin are sparse. Tetracyclines, a group of antibiotics with renal and biliary excretion^56^, were the antibiotic class with the fourth-largest number of species-level associations. These antibiotics are considered to be broad-spectrum due to their activity against Gram-positive bacteria, Gram-negative bacteria and anaerobes; however, their activity within these groups varies because of widespread resistance^57–59^. Macrolides, despite targeting the 50S ribosomal subunit similar to clindamycin and with reported links to gut microbiome composition in another study^60^, showed fewer associations with species abundances. This difference could be due to the stronger activity of clindamycin against anaerobes compared with macrolides^58^ or to the reduced statistical power due to the relatively infrequent use of macrolides in our study^60^. Individuals who frequently use antibiotics also use other medications that can affect the gut microbiome^26,29,30,34^. Many non-antibiotic medications have been shown to have direct antibacterial effects on gut microbes in vitro^32^. Thus, we adjusted our full model for several medications previously linked to gut microbiome composition, as well as polypharmacy^26,29–31,34,60^.

The NPDR captures all antibiotics and prescription medications dispensed in Sweden to outpatients. Because antibiotics provided abroad or during hospitalization are not captured by the register, underestimation of antibiotic use could have affected our results. However, sensitivity analyses after exclusion of participants hospitalized in the 8 years before sampling provided results similar to those of the full model. Another limitation is that our extract from the NPDR does not include treatment indications, which hampered analyses to distinguish the effect of antibiotics from the effects of infections. However, because antibiotics are mainly prescribed to outpatients for airway, urinary tract, skin and soft tissue infections^61^, the possible effect of these infections on the gut microbiome is likely less pronounced than the effect of the antibiotics. Stronger evidence for causality could be achieved from a longitudinal study with sampling before and after the antibiotic course or from a randomized trial. SCAPIS is currently conducting its 8–10-year follow-up, where a second fecal sample is being collected from a subset of participants, which will allow for a stronger study design with repeated sampling.

A prospective study including 36,429 women from the Nurses’ Health Study showed that women with a longer duration of antibiotic use during adulthood had a higher risk of incident cardiovascular disease^5^. A nationwide study using Danish registers found a dose-dependent increased risk of incident IBD, particularly within 1–2 years after antibiotic use^47^. Type 2 diabetes^4^ and CRC^7^ have also been associated with previous antibiotic use. Whether all these associations are due to gut microbiome disruption is yet to be determined. Our study extends this line of research by showing that past antibiotic use is associated with the abundance of species linked to cardiometabolic status, CRC and IBD. The primary reason for a restrictive use of antibiotics is the risk of resistance development. Still, our study adds another argument for reducing antibiotic use: namely, gut microbiome alterations that may persist for many years. As the microbiome field advances, our understanding of the long-term impact of antibiotic treatment beyond infections and resistance may reveal additional health implications.

The main strengths of this study are the large study sample, the comprehensive adjustment for confounders, such as relevant medications and comorbidities, and the fecal metagenomic data from three population-based cohorts profiled using the same method, which allowed harmonization of species annotations and meta-analyses. Our study has limitations. The exact date of fecal sample collection was not systematically recorded, so instead we used the date when the participant visited the test site. However, this should not have affected the long-term associations identified. Although analyses were conducted separately by cohort and followed by meta-analysis, the influence of methodological differences in DNA extraction and sequencing cannot be fully excluded. However, there was no clear evidence for such a substantial influence, because most of the regression coefficients had low heterogeneity, with an estimated meta-analysis *I*^2^ of zero (Supplementary Table 9). A lower detection efficiency of certain species in one of the cohorts could have diluted the signal for those species and biased the estimates toward the null. Furthermore, the generalizability of the findings might be limited to countries with similar antibiotic prescription practices and gut microbiome profiles. Antibiotic use in Sweden is notably restrictive. Certain antibiotics, such as combinations of penicillins with beta-lactamase inhibitors, are rarely prescribed. We had limited statistical power for these antibiotics, and the absence of associations should not be interpreted as an absence of effect. In addition, the high antimicrobial resistance observed in certain populations may render the microbiome more resilient to antibiotic-induced perturbations. Sweden has comparatively low levels of antimicrobial resistance^61^. Differences in antibiotic doses and duration of treatment are likely to affect the gut microbiome but were not investigated in this study. Other limitations include that the Charlson Comorbidity Index derived from hospital records may not capture conditions primarily managed in primary care. Additionally, the information on non-antibiotic medication use lacked dosage details and was limited to the 12 months preceding sampling. Consequently, we did not account for any potential residual effects from medications discontinued before that period, although the residual long-term impact of non-antibiotic drugs on the gut microbiome remains largely underexplored. Further, the abundance of microbiome species was measured as relative abundances, the data type most commonly available in large population-based studies. Relative abundance might not reflect changes in the absolute abundance^62^. Antibiotic residues in food, especially dietary meat, could be a source of antibiotic exposure not accounted for in this study. However, Sweden is noted for its strict policies on antibiotic use in livestock^63^.

In conclusion, we found evidence supporting that certain oral antibiotic classes may influence the gut microbiome composition for more than 4 years. Clindamycin, fluoroquinolones and flucloxacillin had the largest effects. Our results may inform future guidelines on outpatient antimicrobial stewardship interventions and practices, which should, when possible, prioritize antibiotics that have a lower impact on the gut microbiome.

## Methods

### Study population

The study population included participants from the population-based cohorts SCAPIS^22^, SIMPLER^23^ and MOS^24^.

### SCAPIS

SCAPIS enrolled 30,154 women and men aged 50–65 invited from a random sample of residents in areas adjacent to six academic hospitals in Sweden between 2013 and 2018^22^. Participation in SCAPIS entailed two visits to the test sites. In the Uppsala and Malmö sites, participants were provided with material and instructions to collect a fecal sample at home close to the second visit, store it in the home freezer and bring the sample at the second visit. The median interval between visits was 9 days. Participants who did not provide a fecal sample at the second visit were allowed to deliver it later. The date of sampling was not consistently recorded. In total, 9,159 fecal samples were delivered to the test center by the second visit, 244 were delivered ≤7 days after the second visit, and additional 165 samples were delivered >7 days after the second visit. The delivery date was missing for 248 samples, which were assumed to have been delivered at the second visit. Participants also answered an extensive questionnaire on lifestyle, diet and health history. Data on sex were obtained from the Swedish population register. Blood samples were collected and anthropometric measurements were conducted at the test centers. Non-antibiotic medication use was derived from NPDR. PPIs are also sold without a prescription in Sweden, but in smaller packages and for a higher price. Therefore, it can be assumed that most long-term PPI users have a prescription. Besides self-reported doctor diagnoses, the diagnoses of chronic obstructive pulmonary disease, chronic bronchitis and emphysema were attributed to participants with the ICD-10 codes J41, J42, J43 and J44, respectively, in the National Patient Register.

### SIMPLER

The Swedish Mammography Cohort, initiated in 1987, and the Cohort of Swedish Men, initiated in 1997, are two prospective cohorts in central Sweden that constitute SIMPLER^23^. All women identified in the Swedish population register living in Uppsala and Västmanland counties between 1987 and 1990 and born between 1914 and 1948 were invited to the Swedish Mammography Cohort. Similarly, men identified in the population register who were living in Västmanland and Örebro counties in 1997 and born between 1918 and 1952 were invited to participate in the Cohort of Swedish Men^23^. In total, 2,843 women and 3,046 men provided fecal samples between 2012 and 2018. Because fecal samples were analyzed together, using the same procedures, and the phenotype data had been harmonized by SIMPLER, we pooled data from the two cohorts. Participants received via mail the material and instructions to collect a fecal sample at home close to the date of the health examination at the test center. At the time of fecal sampling, participants answered a questionnaire that included questions on smoking and physical activity and a food frequency questionnaire. In cases when smoking was not reported, the most recent smoking information was used, which could be the questionnaire in 2009 or 2019. Anthropometric measurements were performed at the health examination. The comorbidities diagnoses were obtained using the National Patient Register. Medication use was defined as a prescription in the NPDR in the year before fecal sampling.

### MOS

MOS^24^ includes the adult children and grandchildren of the population-based Malmö Diet and Cancer-Cardiovascular Cohort participants^64^. The recruitment occurred between 2013 and 2021. After the exclusion of 39 participants who were also part of SCAPIS, the present study included 2,223 participants enrolled until April 2017 whose fecal samples were analyzed with shotgun metagenomics and passed quality control. Information on comorbidities was self-reported. As in SCAPIS, participants in MOS were instructed to collect the fecal samples at home with the material provided and bring the sample to the test center on the second visit. Participants who did not provide a fecal sample at the second visit could provide a sample at a later time. Similar to SCAPIS, the date of sampling was not consistently recorded in MOS. In total, 1,573 samples were delivered by the second visit, 73 were delivered in the 7 days following the second visit, and 10 were delivered >7 days after the second visit. For the 567 samples where the delivery data was missing, the date of the second visit was used as the delivery date. Lifestyle, diet, health history and comorbidities were assessed using questionnaires. Sex information was retrieved from the Swedish population register. Anthropometric measurements and blood sample collection were performed during the visit to the test center. Medication use was defined as a prescription in NPDR in the last year or self-reported use in the latest week^29^. The retrieval of antipsychotic use information from the NPDR was not included in the ethical approval. Therefore, information on antipsychotic use was based exclusively on self-reports.

### Exclusion criteria

The exclusion criteria included a test center visit before 1 July 2013 (that is, incomplete history of antibiotic use in the past 8 years), an antibiotic prescription in the 30 days before the test center visit, use of antibiotics to treat acne/rosacea or as prophylaxis for urinary tract infection at the time of fecal sampling (long-term antibiotic user), and diagnosis of chronic pulmonary disease (that is, chronic pulmonary obstructive disease, chronic bronchitis and emphysema) or IBD because these conditions often entail a recurrent need for antibiotics and may substantially alter the gut microbiome^65,66^. In SCAPIS, 25 participants who did not consent to the data linkage with registry data were excluded. Because the visit dates were available but not the exact date of fecal sampling, we excluded SCAPIS and MOS participants who (1) had an antibiotic prescription between the two visits, (2) provided fecal samples >7 days after the second visit or (3) had an interval of >60 days between visits, given the uncertainty about sample collection date.

Long-term medications are typically dispensed for three-month periods in Sweden. Therefore, we excluded all participants with a dispensed methenamine prescription (urinary infection prophylaxis) in the three months before the fecal sampling. Likewise, we excluded participants with dispensed nitrofurantoin or trimethoprim prescriptions in the 12 weeks before fecal sampling, summing to at least 22.5 defined daily doses (DDDs), which is equivalent to 50 mg and 100 mg, respectively, once a day for 12 weeks. To exclude long-term users of doxycycline for rosacea, we excluded all participants with a prescription of 40 mg of doxycycline tablets in the 12 weeks before the fecal sampling, all participants with one or more prescriptions of 100 mg of doxycycline tablets adding up to at least 84 DDDs in the last 12 weeks (equivalent to 100 mg per day for 12 weeks), at least 56 DDDs in the last 8 weeks or at least 42 DDDs in last 6 weeks. To exclude long-term users of tetracycline or lymecycline for rosacea, we excluded all participants with dispensed prescriptions adding up to at least 42 DDDs in the last 12 weeks (equivalent to 500 mg per day or 300 mg per day, respectively, for 12 weeks), at least 28 DDDs in the last 8 weeks or at least 21 DDDs in the last 6 weeks. The number of individuals excluded per criterion is provided in Extended Data Fig. 1.

### Antibiotic exposure

All oral antibiotics dispensed to outpatients in Sweden require a prescription and are registered in the NPDR^21^. We retrieved information on all dispensed prescriptions with the Anatomical Therapeutic Chemical (ATC) code J01 (antibacterials for systemic use) and classified them as tetracyclines (J01A), extended-spectrum penicillins (J01CA), beta-lactamase-sensitive penicillins (J01CE), beta-lactamase-resistant penicillins (J01CF), penicillins combined with beta-lactamase inhibitors (J01CR), cephalosporins (J01DB, J01DC, J01DD), sulfonamides and trimethoprim (J01E), macrolides (J01FA), lincosamides (J01FF), fluoroquinolones (J01MA) and nitrofurantoin (J01XE01). In Sweden, amoxicillin with clavulanic acid was the only oral penicillin combination available, penicillin V was the only beta-lactamase-sensitive penicillin, flucloxacillin was the only beta-lactamase-resistant penicillin and clindamycin was the only lincosamide. The only extended-spectrum penicillins were amoxicillin and pivmecillinam. Antibiotic prescriptions were divided into three periods: <1 year, ≥1 and <4 years and ≥4 and <8 years before the fecal sampling.

### Fecal metagenomics

In all three cohorts, participants were instructed to collect the fecal samples at home close to the date of the test center visit and to store the samples in the home freezer until the visit, after which the samples were stored at −80 °C.

SCAPIS and MOS fecal samples were sent to Cmbio (Copenhagen, Denmark) for DNA extraction and shotgun metagenomic sequencing^10^. DNA extraction was performed using NucleoSpin 96 Soil kits. Each round of extraction contained one negative and one positive control. Following DNA fragmentation and library preparation, sequencing was conducted with the Illumina NovaSeq6000 system. The average sequence depth was 25.3 million read pairs for SCAPIS samples from Uppsala and 26.3 million read pairs for SCAPIS samples from Malmö and MOS samples.

The fecal samples from SIMPLER were sent to the Centre for Translational Microbiome Research at the Karolinska Institute in Stockholm, Sweden. Before shipping, the samples were aliquoted into FluidX tubes containing 800 μl of DNA/RNA Shield buffer (R1100-250, Zymo Research). The DNA extraction was conducted with the MagPure Stool kit (Magen Biotechnology Co.) and included a bead-beating step in a FastPrep-96 at 1,600 rpm for 1 minute. One negative and one positive control were added to each batch. Library preparation was performed using the MGIEasy FS DNA Library Prep Set kit. The libraries were sequenced in MGI Tech Co (Latvia) using DNBseq 2 × 100 bp paired-end sequencing on the DNBSEQ G400 or T7 sequencing instrument (MGI Tech Co.). The average sequence depth was 51 million read pairs.

The metagenomic reads from all three cohorts were profiled by Cmbio using its Human Profiler (CHAMP) and delivered as relative abundance of species^67^. Briefly, reads that mapped to the human reference genome GRCh38.p14 were removed using Bowtie2 (v 2.4.2). The non-host reads were mapped to the Cmbio HMR05 gene catalog^67^ using BWA mem (v. 0.7.17). The relative abundance of each species was calculated based on the signature genes with observed read counts within the expected 99% quantile and normalized sample-wise so that the total abundance of all species summed to 100%. The expected 99% quantile of read counts was calculated for each gene based on a negative binomial distribution with a mean proportional to the effective gene length and dispersion as log2(effective gene length). The taxonomic annotation of species was performed using the Genome Taxonomy Database release 214^68^.

A rarefied species relative abundance table was produced by random sampling, without replacement, of 190,977 gene counts per sample in SCAPIS and MOS and 641,964 gene counts in SIMPLER. The diversity of microbiota species in each sample was assessed using the rarefied table to calculate three alpha diversity metrics: Shannon Index, species richness and inverse Simpson Index. Although richness represents the number of species, the Shannon and inverse Simpson metrics account for both the richness and evenness of the abundances. All other analyses were conducted with the non-rarefied relative abundance table.

The relative abundance of species was subjected to centered log-ratio transformation after addition of a pseudo-value equal to the minimal non-zero value. After transformation, the values that were zero before transformation were replaced with the minimal non-zero transformed value per species, preventing the initial zeros from having different transformed values for the same species. We kept for subsequent analysis the 1,340 species present in >2% of the participants in the three cohorts.

For SCAPIS and MOS data, DNA extraction plate was the technical variable most strongly associated with Shannon diversity and the first 10 principal coordinates of Bray–Curtis dissimilarity. For SIMPLER, aliquoting plate was the most strongly associated technical variable. Therefore, adjustment for these variables was recommended to control for within-cohort batch effects in statistical analyses.

### Statistical analyses

Our assumptions about the effects of temporally stable covariates on our exposure and outcomes were displayed on a basic model DAG^25^ (Supplementary Fig. 3a). The d-separation criteria were applied to the DAG to select covariates for model adjustment. Some potentially time-varying covariates were primarily collected at the time of fecal sampling and thus after the antibiotic exposure. A full-model DAG (Supplementary Fig. 3b) was created to account for comorbidities and medications that may affect the gut microbiome. Here, we included BMI, the Charlson Comorbidity Index, polypharmacy and medication use. The Charlson Comorbidity Index includes the diagnosis of diabetes, cancer and rheumatologic, cardiovascular, liver and renal diseases^28^. The index was derived from extracts from the National Patient Register^27^ since 1970 in SCAPIS and 1967 in SIMPLER. In MOS, the index was constructed using extracts from the National Inpatient Register since 1997 and from the Outpatient Register in the Region Skåne in 1997–2000. Because non-complicated type 2 diabetes is primarily treated in primary care centers and not included in the National Patient Register, self-reported diabetes was also used in SCAPIS and MOS and metformin use (ATC A10BA02) based on NPDR in SIMPLER. Polypharmacy in SCAPIS and SIMPLER was defined based on the NPDR as the dispensed prescription of ≥5 medications for regular use. A medication was considered to be in regular use if it had been dispensed ≥3 times in the last 12 months. Medications in Sweden are typically dispensed for periods of three months. In MOS, polypharmacy was based on self-reported use of ≥5 medications in the latest week, as previously described^29^.

The basic model included age, sex, education, smoking and country of birth. Test-site-specific analysis plates were also included to account for technical variability. The full model additionally included BMI, polypharmacy, the Charlson Comorbidity Index and use of PPIs (ATC: A02BC), metformin, SSRIs (N06AB), statins (C10AA), beta-blockers (C07AB) and/or antipsychotics (N05A) within 1 year before fecal sampling.

For the multivariable regression models, microbiome species diversity metrics and the abundance of each species were modeled individually as the dependent variable. The number of courses of each antibiotic class in the three periods (<1 year, 1–4 years, 4–8 years) before fecal sample collection were included as independent variables in the same model:

$$\begin{array}{ll}{\mathrm{diversity}\,\mathrm{or}\,\mathrm{species}}_{\mathrm{clr}} & ={\mathrm{abx}1}_{4-8{\rm{y}}}+{\mathrm{abx}1}_{1-4{\rm{y}}}+\mathrm{abx}{1}_{ < 1{\rm{y}}}\\ & +{\mathrm{abx}2}_{4-8{\rm{y}}}+\ldots +{\mathrm{abx}11}_{ < 1{\rm{y}}}+\mathrm{covariates}\end{array}$$

where abx1*–*abx11 are the number of courses of the 11 antibiotic classes, diversity is the gut microbiome species diversity, and species_clr_ is the centered log-ratio–transformed species abundance. The data from each cohort were analyzed separately and then subjected to inverse-variance weighted fixed-effects meta-analyses (R package metafor v.4.4.0). Linear regression models were used for SCAPIS and SIMPLER; linear mixed-effects models (R package lmerTest v.3.1.3) with family as a random intercept were used for MOS because this study had a family-based recruitment of participants. The generalized variance inflation factor (R package car v.3.1.2) was calculated to determine whether collinearity affected models.

To estimate the marginal mean of the gut microbiome species diversity associated with each additional antibiotic course within each period, we counted the number of prescriptions for all antibiotics in each period and modeled them as independent variables using restricted cubic splines with three knots and adjusted for the full-model covariates. The estimated marginal means (EMMs) were weighted-averaged across levels of categorical variables, and continuous variables were set to their mean. Pairwise comparisons of the EMMs between different numbers of antibiotic courses were made using the function ‘pairs’ from the R package emmeans (v.1.8.8).

All statistical analyses used two-sided *P* values. Multiple testing was accounted for using the Benjamini–Hochberg method^69^; an FDR of 5% (*q-*value < 0.05) was considered significant. For antibiotic–species associations that were significant in the full-model meta-analysis (*q-*value < 0.05) and showed heterogeneity (Cochran’s *Q* *P* value < 0.05), we assessed whether any single individual influenced the result. Within each cohort, we reran the linear regression and calculated dfbetas for the antibiotic exposure with a *q-*value < 0.05. The observation with the highest dfbeta in each cohort was removed, the models were refitted, and the cohort-specific estimates were meta-analyzed again. If the updated meta-analysis had a *P* -value > 0.05, the original association was considered non-robust and discarded.

In the main analyses, participants were excluded if they had used antibiotics within 30 days prior to the test center visit. To assess the robustness of this criterion, we conducted sensitivity analyses by repeating the analyses using different exclusion periods: no exclusion for recent antibiotic use, and exclusions for antibiotic use within 6 months or 12 months before fecal sampling.

To assess whether our full model sufficiently controlled for confounders, we used as a negative control exposure the association between antibiotic use in the year after fecal sampling and the gut microbiome species diversity, either adjusting for antibiotic use before the sampling or restricting to participants with no antibiotic use in the 8 years before the sampling. We excluded 1,463 participants in SCAPIS for whom register data were unavailable for the year after fecal sampling. We performed two additional sensitivity analyses in SCAPIS and in SIMPLER because data about hospitalization from the National Patient Register were available. In the first, participants who had been hospitalized for a condition likely to be treated with antibiotics (Supplementary Table 15) were excluded. In the second, participants who had been hospitalized for any reason in the previous 8 years were excluded.

We explored potential sex and age differences by conducting stratified analyses for visualization and fitting interaction terms for inference. In the age-stratified analysis, participants were grouped into ≤55 and >55 years at fecal sampling. All SIMPLER participants were >55 years; thus, all participants from this cohort were included in the older stratum. When ≤5 individuals within a stratum of a cohort were exposed to a given antibiotic in a specific period, that antibiotic-period term was excluded from the model for that stratum of the cohort. This occurred especially in MOS for sulfamethoxazole-trimethoprim, amoxicillin-clavulanic acid, macrolides and nitrofurantoin in men. As in the main analyses, the regression models were fitted separately in each cohort, and the resulting estimates were meta-analyzed. For each antibiotic, we conducted a series of regression models including age or sex interactions with the three antibiotic-period variables. We then applied the likelihood-ratio test comparing models with and without the interaction terms. The likelihood-ratio test *P* values from the three cohorts were meta-analyzed using Fisher’s method. A significant meta-analyzed *P* value after multiple testing correction was interpreted as evidence of interaction. Age was modeled as a continuous variable to allow the inclusion of SIMPLER in this analysis. For the sex interaction, >5 men and >5 women within a cohort had to be exposed to the antibiotic in all three periods for the antibiotic to be evaluated in that cohort. In the age interaction analyses, >10 individuals in the cohort had to be exposed to the antibiotic in all three periods.

To explore the link between species and cardiometabolic markers, we used data from the largest cohort, SCAPIS. The cardiometabolic markers were BMI, WHR, systolic blood pressure, serum TG, non-high-density lipoprotein cholesterol, HbA1c and high-sensitivity CRP. Partial Spearman correlations (R package ppcor v.1.1) were estimated between the markers and the species relative abundances, adjusting for age, sex, country of birth, education, smoking, site-specific analysis plate and BMI (except when BMI was the marker of interest). The 743 individuals with diabetes were removed, given the strong link of this condition to gut microbiome and cardiometabolic disease.

### Functional regression model

A functional regression model with scalar response was implemented using the fda (v6.1.4) and fda.usc (v.2.1.0) R packages and using monthly resolution of antibiotic exposure. For each month before fecal sampling, participants were categorized as users or non-users of each antibiotic class in that month. In a conventional standard linear regression model, this would produce unstable estimates due to the large number of predictor variables. However, the functional regression model uses the high correlation between temporally adjacent regression coefficients by fitting a cubic spline. Models were fitted for each of the three cohorts separately, using the same covariates as in our full-model analysis. The antibiotic classes cephalosporins, macrolides, amoxicillin-clavulanic acid, sulfamethoxazole-trimethoprim and nitrofurantoin were combined into a single class to increase the model stability as they occurred infrequently in the data. Pointwise standard errors for each monthly estimate were estimated using bootstrap. Finally, the estimates and standard errors of each month were combined using a fixed-effects meta-analysis. Estimates and 95% confidence intervals (assuming approximate normality of estimates due to the central limit theorem) were plotted for each class of antibiotics.

All analyses used R version 4.3.2. Because the number of missing data was small, a complete case analysis was performed (Extended Data Fig. 1).

### Associations with CRC and IBD

We identified species associated with CRC and IBD from two previous case–control studies. Akiyama et al. examined the gut microbiome in 31 patients with Crohn’s disease, 111 with ulcerative colitis and 540 controls from the Japanese 4D cohort^48^. Fecal samples were analyzed by shotgun metagenomic sequencing and taxonomically profiled with mOTUs v3.0.1. Associations between IBD and microbial species were assessed using regression models adjusted for age, sex and BMI. Piccinno et al. investigated CRC by integrating data from 18 studies comprising 1,472 CRC cases and 1,568 controls^14^. Samples were profiled with MetaPhlAn 4, and species associations were tested with regression models adjusted for the same covariates. For comparison with our results, we included only taxa with species-level annotation. In total, 65 CRC- and 56 IBD-associated taxa were identified, of which 37 (CRC) and 39 (IBD), representing 59 unique taxa, could be mapped to species or subspecies present in our study.

### Ethics committee approval

Ethical approval was obtained from the Swedish Ethical Review Authority (DNR 2018-315 B and amendments 2020-06597 and 2022-06460-02, DNR 2012-594 and amendments 2017-768 and 2020-05611, DNR 2022-06137-01 and amendment DNR 2023-04785-02). All participants in the three cohorts provided written informed consent.

### Reporting summary

Further information on research design is available in the Nature Portfolio Reporting Summary linked to this article.

## Online content

Any methods, additional references, Nature Portfolio reporting summaries, source data, extended data, supplementary information, acknowledgements, peer review information; details of author contributions and competing interests; and statements of data and code availability are available at 10.1038/s41591-026-04284-y.

## Supplementary information

Supplementary Information Supplementary Figs. 1–12.

Reporting Summary

Supplementary Tables Supplementary Tables 1–15.

## Data Availability

The data supporting the conclusions of this article were provided by the SCAPIS, SIMPLER and MOS data offices and contain sensitive personal information protected under privacy laws; therefore, they are not publicly available. Requests for access to data to verify the analyses and findings of this study should be directed to the corresponding author. An initial response to the request will be provided within two weeks. Data will be shared once a data-sharing agreement has been signed between Uppsala University and the requestor’s institution, and following approval from the Swedish Ethical Review Authority (https://etikprovningsmyndigheten.se) and the boards of SCAPIS, SIMPLER and MOS. Requests for data access for additional research purposes should be directed to the respective cohorts: SCAPIS (https://www.scapis.org/data-access/), SIMPLER (https://www.simpler4health.se/w/sh/en/researchers/data-access) and MOS (https://www.malmo-cohorts.lu.se/application-data-and-samples/applying-samples-mdc-and-mpp). Dehosted anonymized metagenomic sequencing data from SCAPIS are available in the European Nucleotide Archive under accession number PRJEB51353.

## Code availability

The code used for the statistical analysis and for generation of figures and tables is available at https://github.com/MolEpicUU/antibiotic_gut.
