# Supplementary material for: Antibiotic use and gut microbiome composition links from individual-level prescription data of 14,979 individuals
Source: Nat Med. 2026 Mar 11;32(4):1351–61. doi: 10.1038/s41591-026-04284-y (PMC13099378; doi:10.1038/s41591-026-04284-y)
Supplement: Supplementary file 1 — Supplementary Figs. 1–12. [file 41591_2026_4284_MOESM1_ESM.pdf]

nature medicine

Supplementary information

https://doi.org/10.1038/s41591-026-04284-y

Antibiotic use and gut microbiome composition links from individual-level prescription data of 14,979 individuals

In the format provided by the authors and unedited

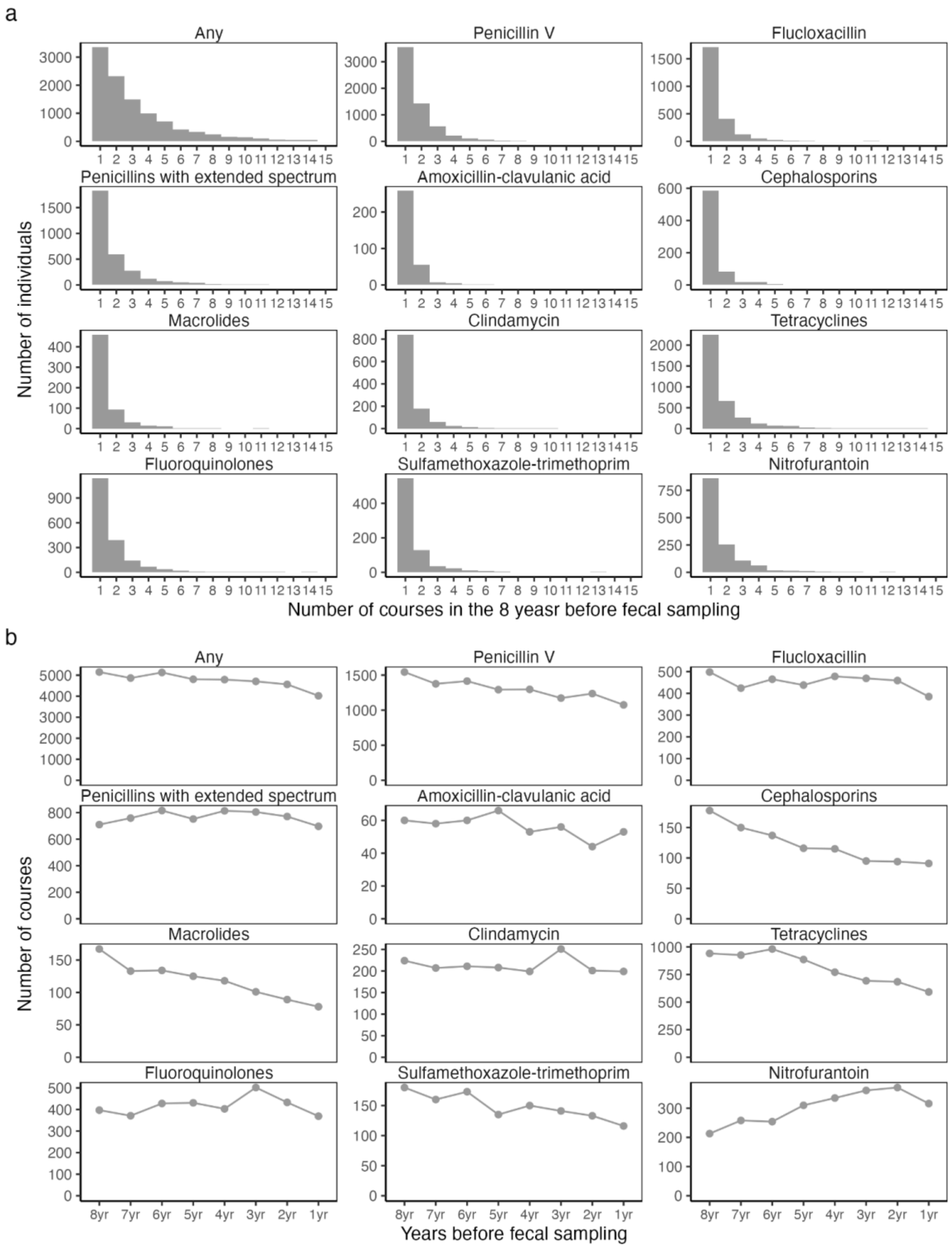

Supplementary Figure 1. Frequency and temporal trends of antibiotic prescriptions in the 8 years before sampling in SCAPIS, SIMPLER, and MOS combined. a. Histograms showing the distribution of individuals by number of antibiotic courses over the entire 8-year period. Individuals with no antibiotic courses are not included. **b.** Line plots of the number of prescriptions dispensed during each of the 8 years before fecal sampling.

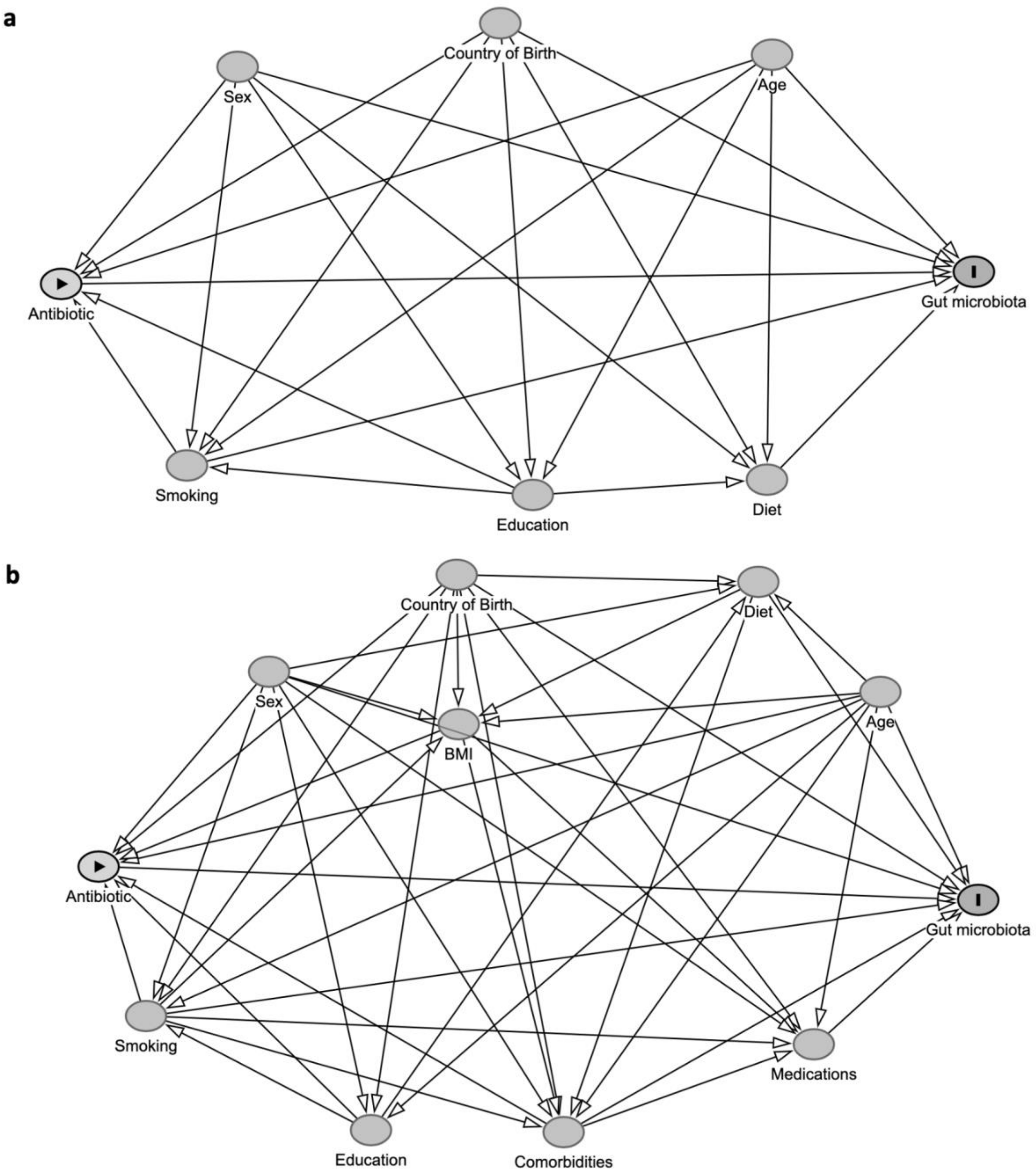

Supplementary Figure 2. Directed Acyclic Graphs. a. Basic model directed acyclic graph of the hypothetical causal diagram of the effect of antibiotic use on gut microbiota, focusing on the temporally stable covariates. **b.** Full model directed acyclic graph

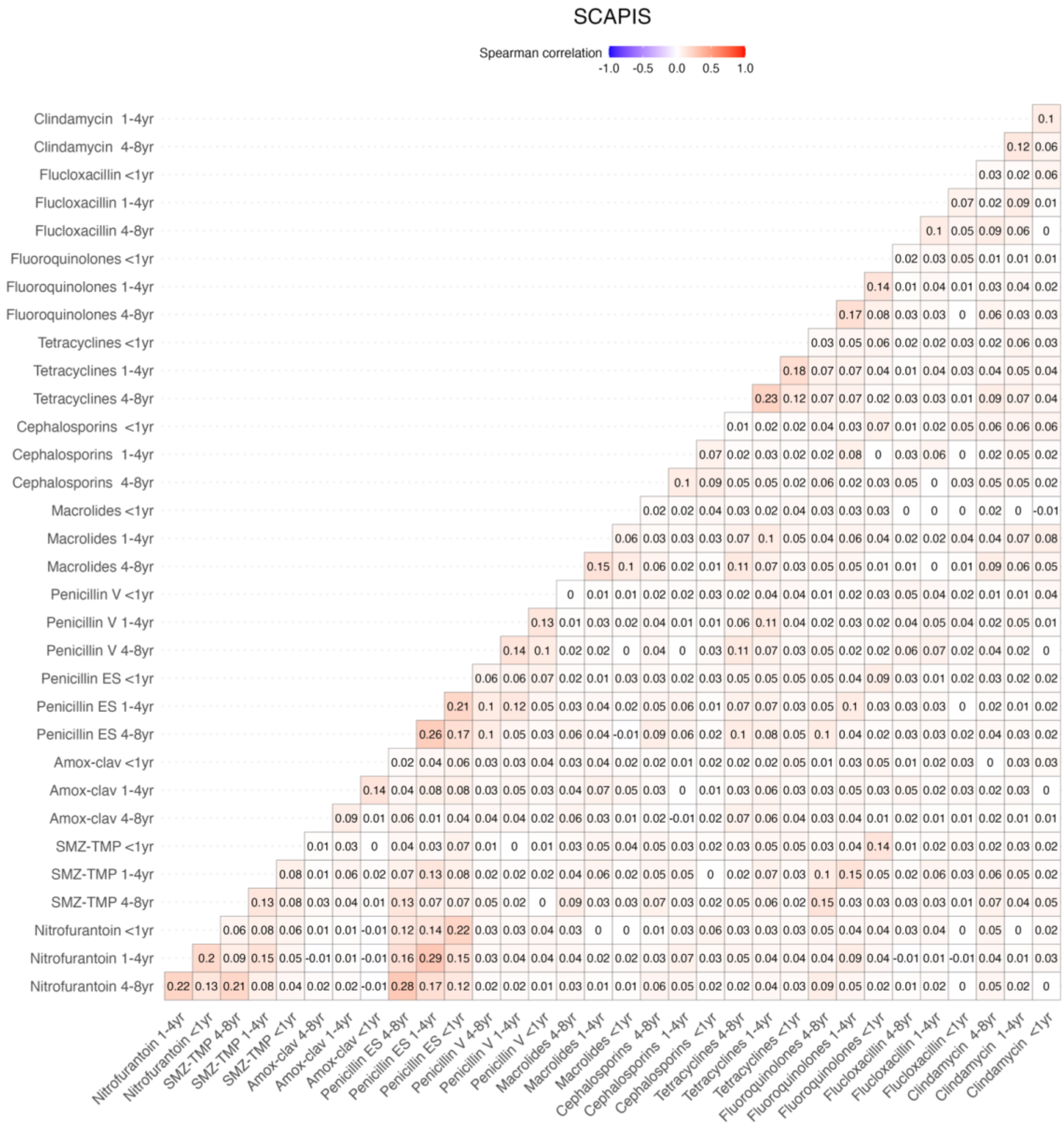

Supplementary Figure 3. Spearman correlation between the number of antibiotic courses of each participant in SCAPIS. Spearman correlation between the number of courses for each antibiotic class in the periods <1 year, 1–4 years, and 4–8 years before the fecal sampling (n = 8,488). Penicillin ES: extended-spectrum penicillins; Amox-clav: amoxicillin-clavulanic acid; SMZ-TMP: sulfamethoxazole-trimethoprim.

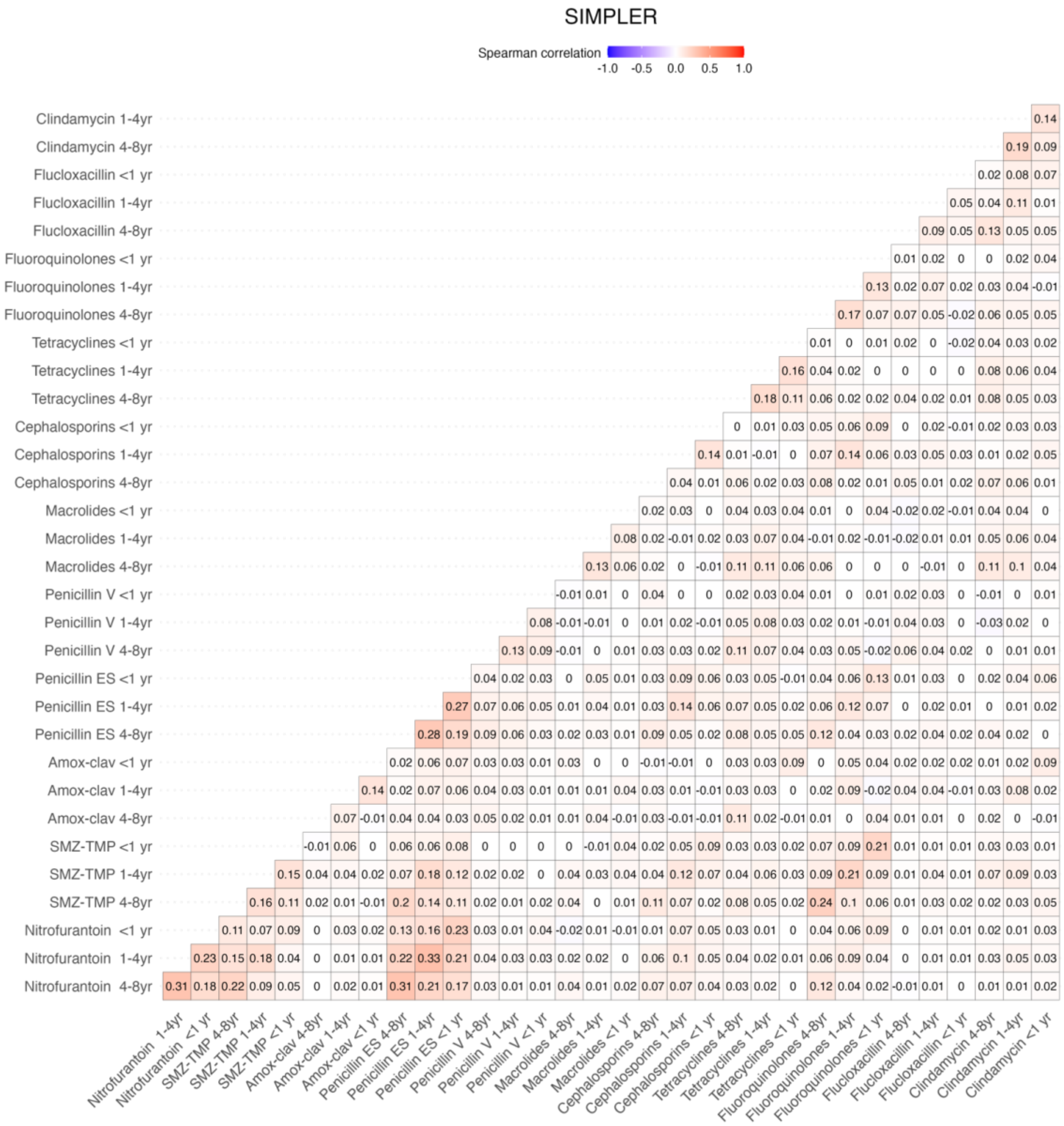

Supplementary Figure 4. Spearman correlation between the number of antibiotic courses of each participant in SIMPLER. Spearman correlation between the number of courses for each antibiotic class in the periods <1 year, 1–4 years, and 4–8 years before the fecal sampling (n = 4,784). Penicillin ES: extended-spectrum penicillins; Amox-clav: amoxicillin-clavulanic acid; SMZ-TMP: sulfamethoxazole-trimethoprim.

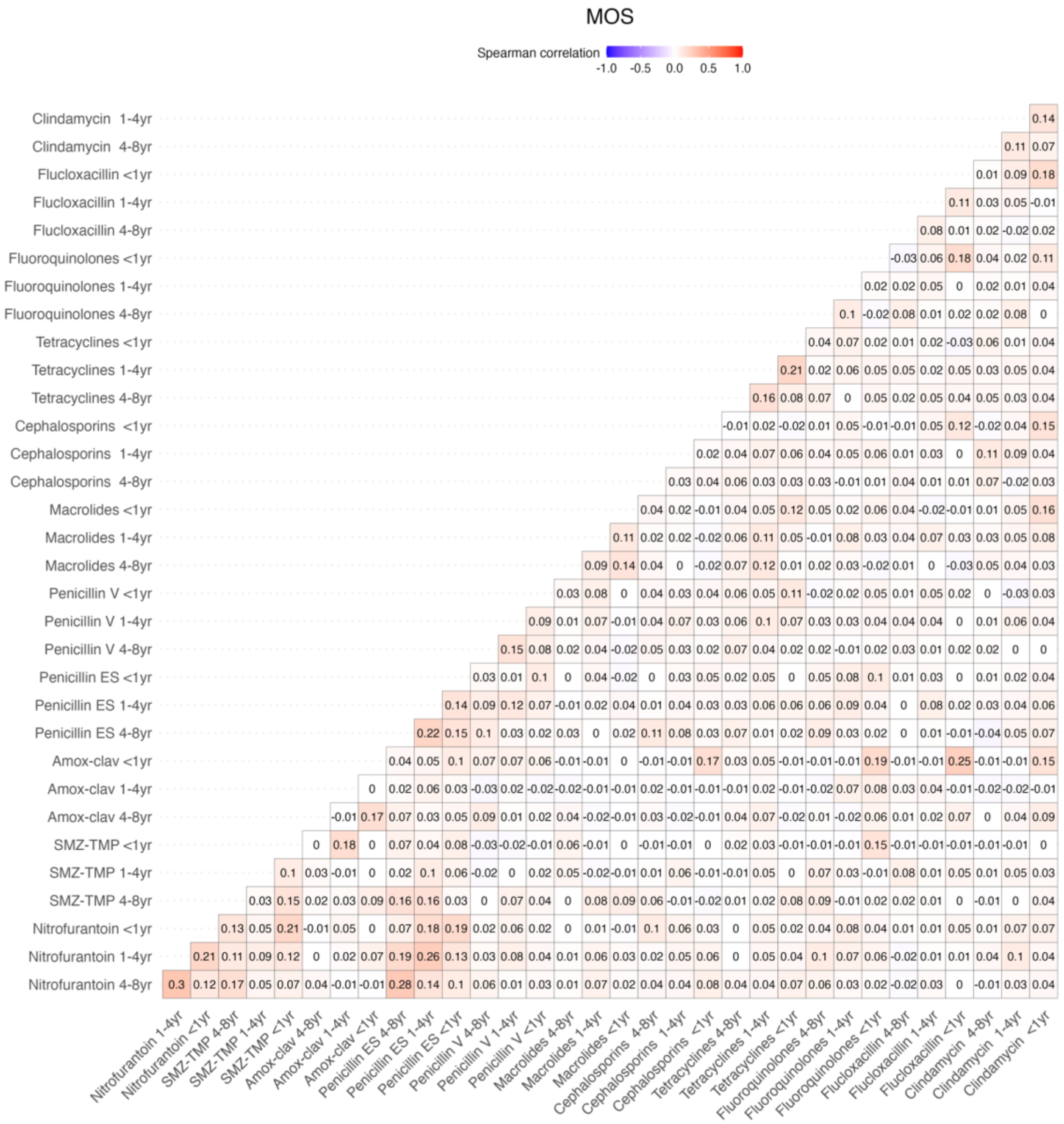

Supplementary Figure 5. Spearman correlation between the number of antibiotic courses of each participant in MOS. Spearman correlation between the number of courses for each antibiotic class in the periods <1 year, 1–4 years, and 4–8 years before the fecal sampling (n = 1,707). Penicillin ES: extended-spectrum penicillins; Amox-clav: amoxicillin-clavulanic acid; SMZ-TMP: sulfamethoxazole-trimethoprim.

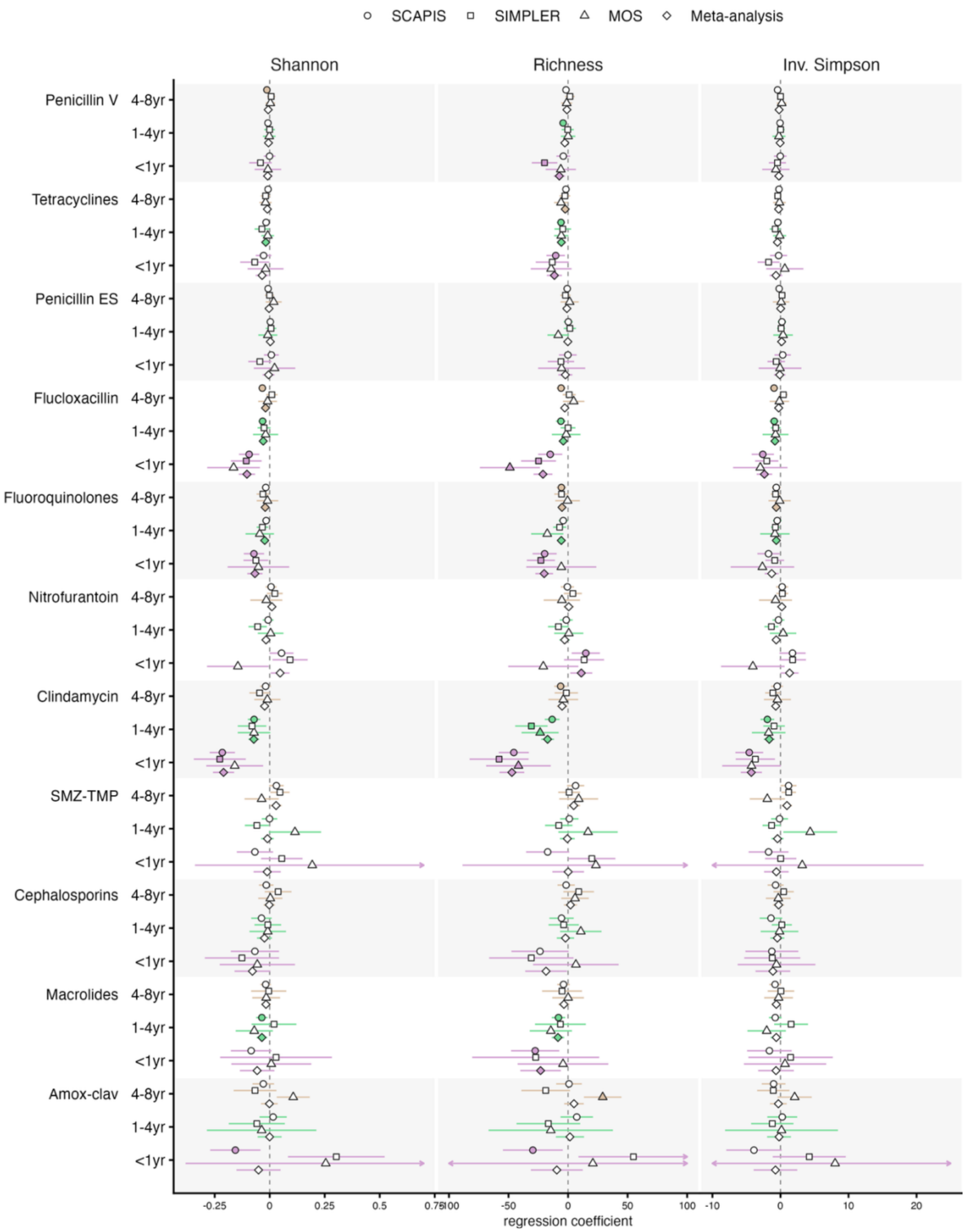

Supplementary Figure 6. Associations between antibiotic use in the 8 years before fecal sampling and gut microbiota species diversity (Shannon index, species richness, and inverse Simpson index) in each study and meta-analyzed. Associations were investigated using regression models adjusted for age, sex, smoking, education, country of birth, site-specific analysis plate, body mass index, Charlson comorbidity index, polypharmacy, and use of proton-pump inhibitors, metformin, selective serotonin reuptake inhibitors, statins, beta-blockers, and antipsychotics. In MOS, linear mixed models were used with family as a random effect. Meta-analyses were performed using fixed-effects models. The x-axis displays the regression coefficients; error bars represent 95% confidence intervals. Filled shapes indicate significant associations (FDR <5%).

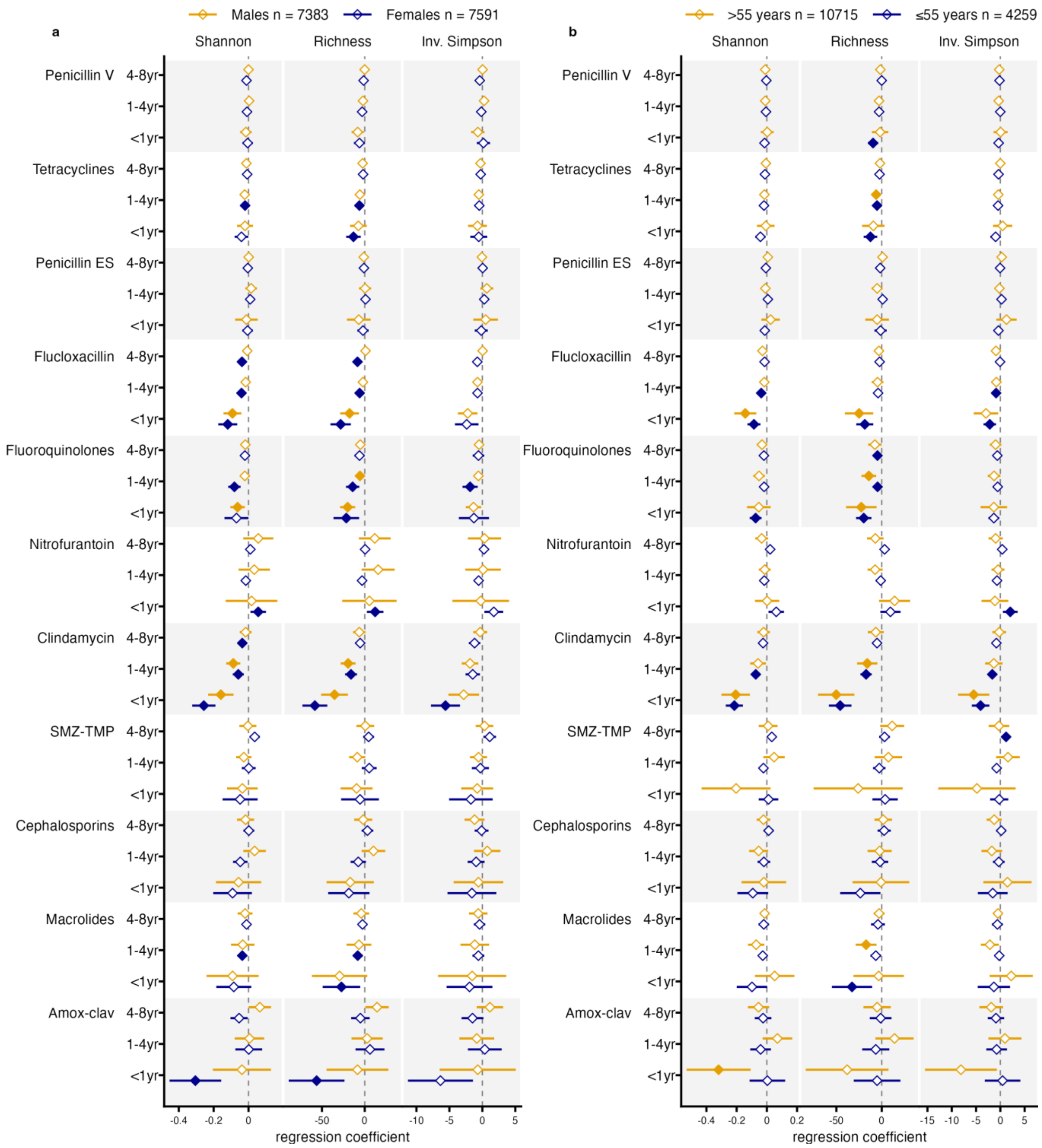

Supplementary Figure 7. Age- and sex-stratified associations with gut microbiome diversity. Associations between antibiotic use and gut microbiome species diversity, stratified by (a) sex or (b) age, were assessed using regression models adjusted for age, smoking, education, country of birth, site-specific analysis plate, body mass index, Charlson comorbidity index, polypharmacy, and use of proton-pump inhibitors, metformin, selective serotonin reuptake inhibitors, statins, beta-blockers, and antipsychotics. Sex was included in the age-stratified analysis. The x-axis and diamond shapes display the meta-analyzed regression coefficients; error bars represent 95% confidence intervals. Filled symbols indicate statistically significant associations (FDR < 5%).

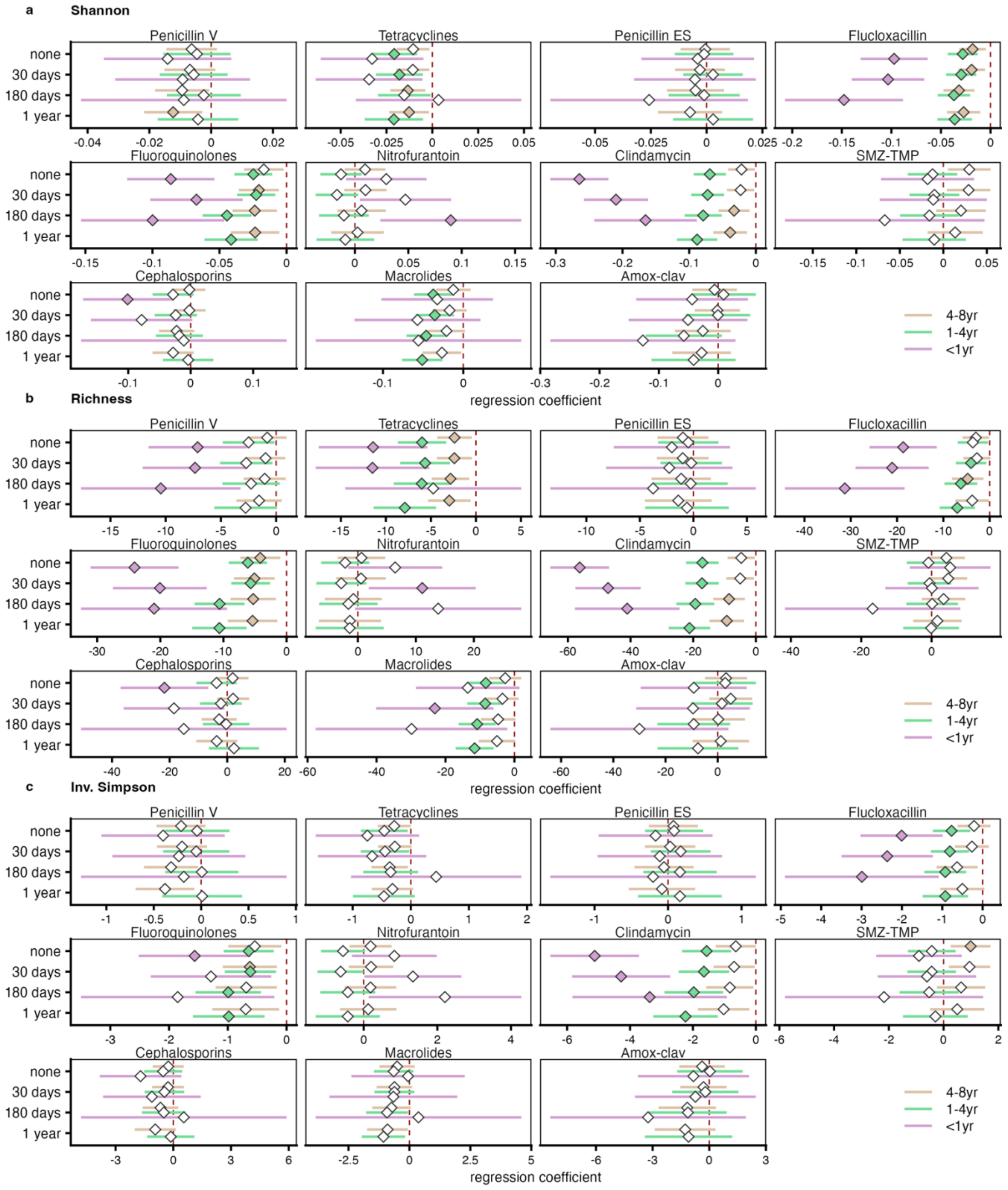

Supplementary Figure 8. Associations between antibiotic use during the 8 years before fecal sampling and gut microbiome species diversity under alternative exclusion windows. Outcomes are (a) Shannon diversity, (b) species richness, and (c) inverse Simpson index. Regression models adjusted for age, sex, smoking, education, country of birth, plate, BMI, Charlson comorbidity index, polypharmacy, and use of proton-pump inhibitors, metformin, selective serotonin reuptake inhibitors, statins, beta-blockers, and antipsychotics. Exclusion windows were: none (n = 15,258), 30 days (n included = 14,974), 180 days (n included = 13,653), and 1 year (n included = 12,350) before sampling. The x-axis and diamonds show meta-analyzed regression coefficients; error bars represent 95% confidence intervals; filled diamonds indicate FDR < 5%.

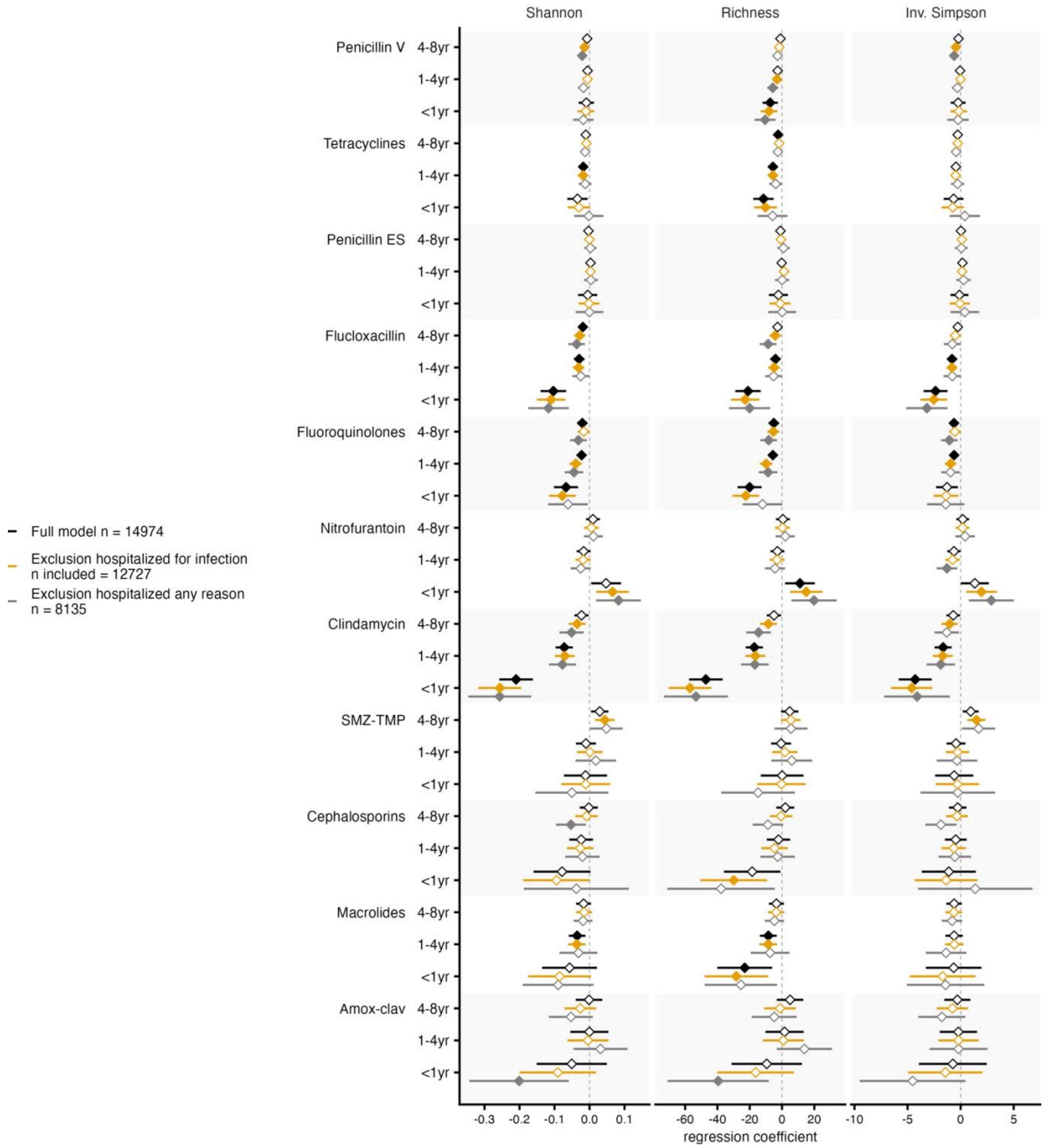

Supplementary Figure 9. Sensitivity analysis on the associations with gut microbiota species diversity. (Shannon index, species richness, and inverse Simpson). For each cohort, we used regression models with the number of prescriptions for each antibiotic class in <1 year, 1–4 years, and 4–8 years before the fecal sampling as main exposures. Two sensitivity analyses were conducted using only SCAPIS and SIMPLER: 1) removal of participants (n=540) hospitalized in the last 8 years due to infection (Supplementary table 15), and 2) removal of participants (n=5,129) hospitalized for any cause. The full model includes the complete study population from SCAPIS, SIMPLER, and MOS. Models were adjusted for age, sex, smoking, education, country of birth, site-specific analysis plate, body mass index, Charlson comorbidity index, polypharmacy, and use of proton-pump inhibitors, metformin, selective serotonin reuptake inhibitors, statins, beta-blockers, and antipsychotics. The x-axis and diamonds display the meta-analyzed regression coefficients; error bars represent 95% confidence intervals; filled diamonds indicate significant associations (FDR <5%).

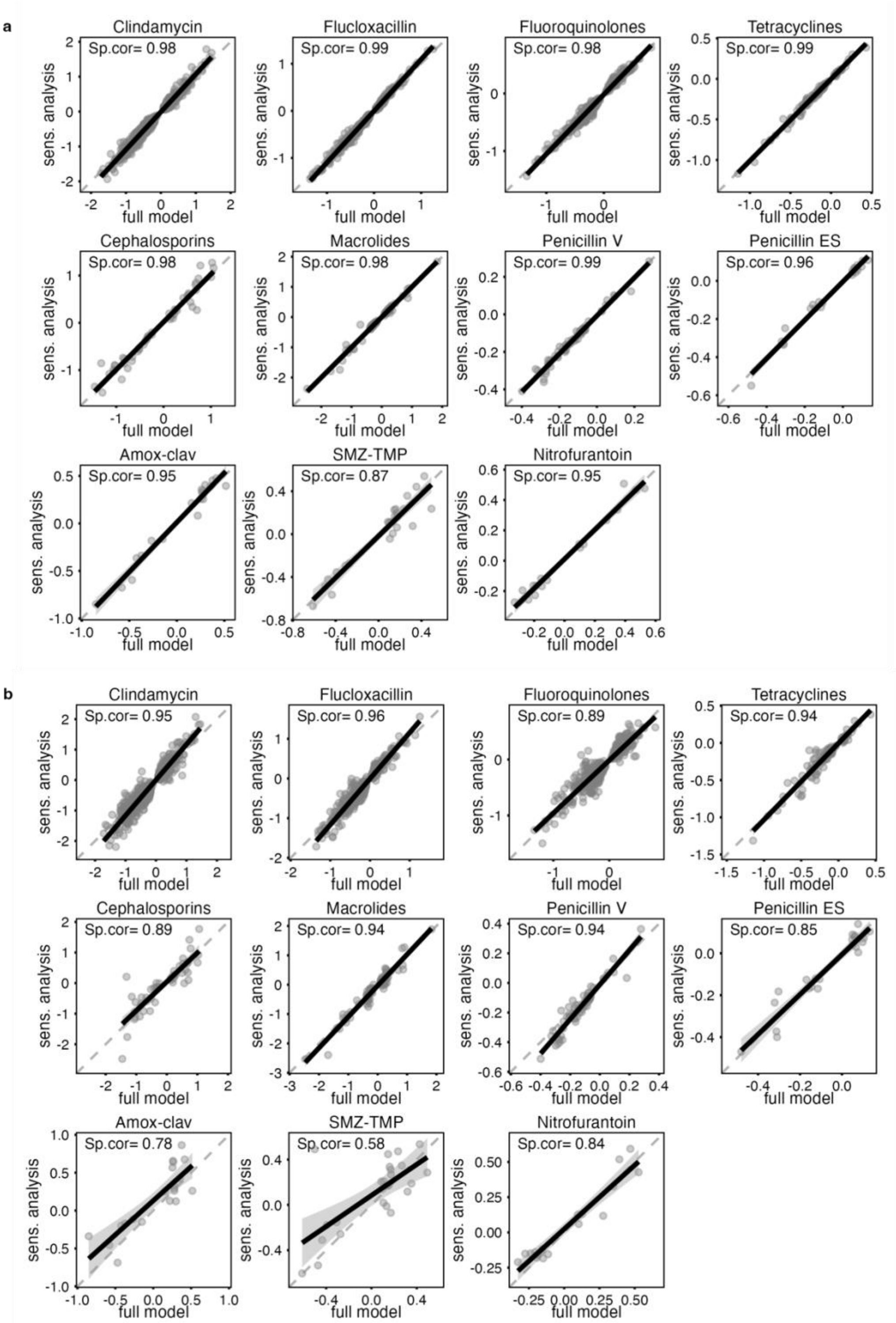

Supplementary Figure 10. Sensitivity Analysis on the Associations with Gut Microbiota Species Abundance. Comparison of meta-analyzed regression coefficients between the full model (n = 14,974) and sensitivity analysis associations for the significant full model associations (FDR <5%). **a.** Associations between antibiotic use in the 8 years before fecal sampling and gut microbiota species abundances were investigated in SCAPIS and SIMPLER after removing 540 individuals who had been hospitalized due to an infection (Supplementary Table 15) in the last 8 years (n included = 12,727), and **b**. after removing 5,129 individuals who had been hospitalized due to any reason in the last 8 years (n included = 8,135). Analyses were performed in each cohort, followed by meta-analyses of the regression coefficients. Models were adjusted for age, sex, smoking, education, country of birth, site-specific analysis plate, BMI, Charlson Comorbidity Index, polypharmacy, and use of proton-pump inhibitors, metformin, selective serotonin reuptake inhibitors, statins, beta-blockers, and antipsychotics. Amox-clav: Amoxicillin-clavulanic acid, Penicillin ES: penicillins extended spectrum; SMZ-TMP: sulfamethoxazole-trimethoprim; Sp. cor = Spearman´s correlation.

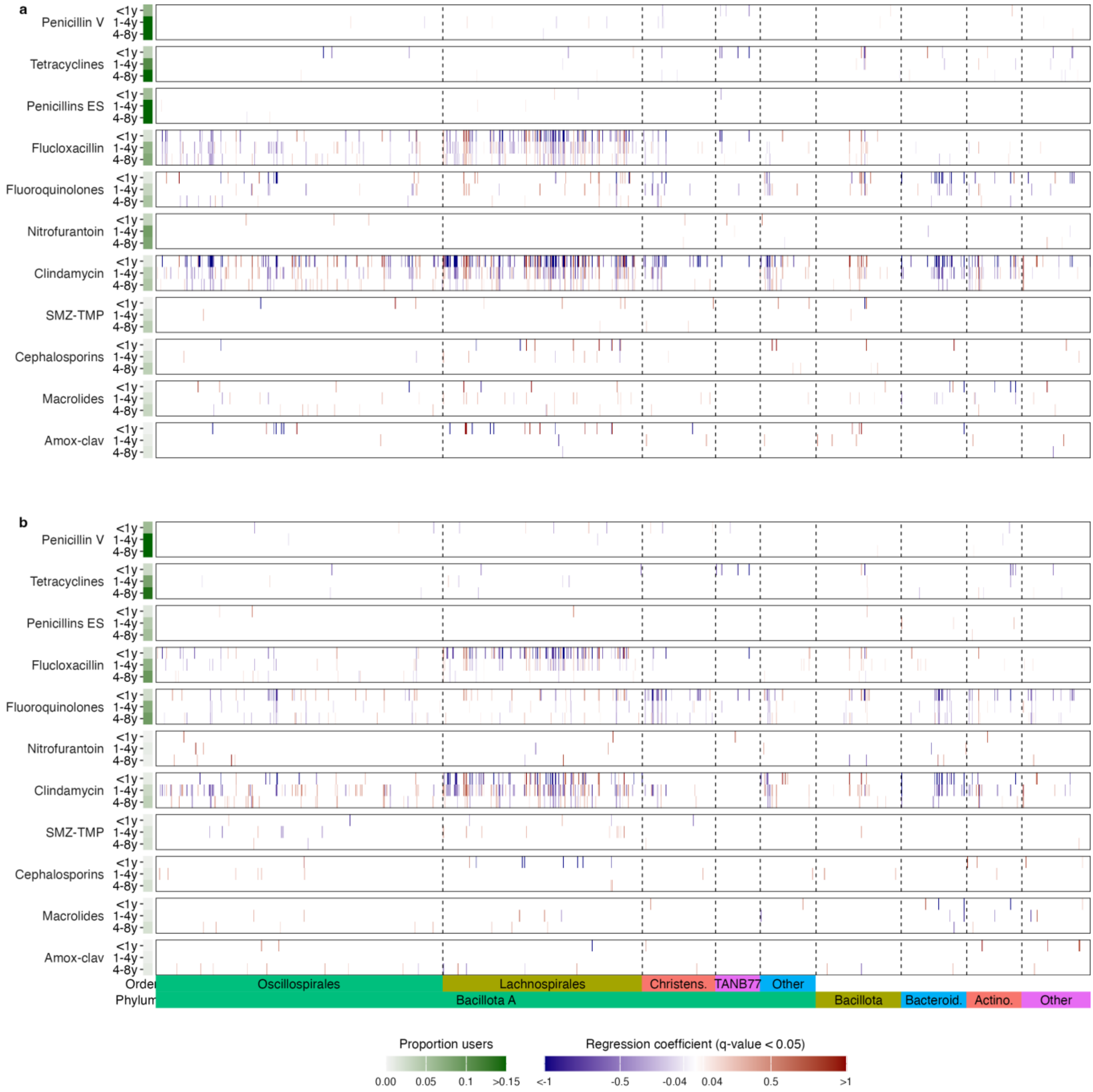

Supplementary Figure 11. Antibiotic use and its associations with abundance of gut microbiome species stratified by sex. a. females (n = 7,591), **b**. males (n = 7,383). The associations between the number of antibiotic courses before fecal sampling and species abundance were investigated using regression models adjusted for full-model covariates (except sex) in three cohorts (SCAPIS, MOS, and SIMPLER, total n = 14,974), followed by meta-analyses. Blue or red vertical bar represents a negative or positive association, respectively. Only significant associations are shown (FDR <0.05). The y-axis displays the 11 antibiotic classes and the periods of the antibiotic courses: <1 year, 1–4 years, and 4–8 years before fecal sampling. The proportion of users indicates the proportion of (**a**) females and (**b**) males who had at least one course of the respective antibiotic in that period. The 1340 species in the x-axis were ordered based on their taxonomy. Phyla are identified at the bottom of the plot. For the phylum *Bacillota* A, the taxonomic orders are also displayed. Penicillins ES: extended-spectrum penicillins; SMZ-TMP: sulphamethoxazole-trimethoprim; Amox-clav: amoxicillin-clavulanic acid; Bacteroid.: *Bacteroidota*; Actino.: *Actinomycetota*; Christens: *Christensenellales.*

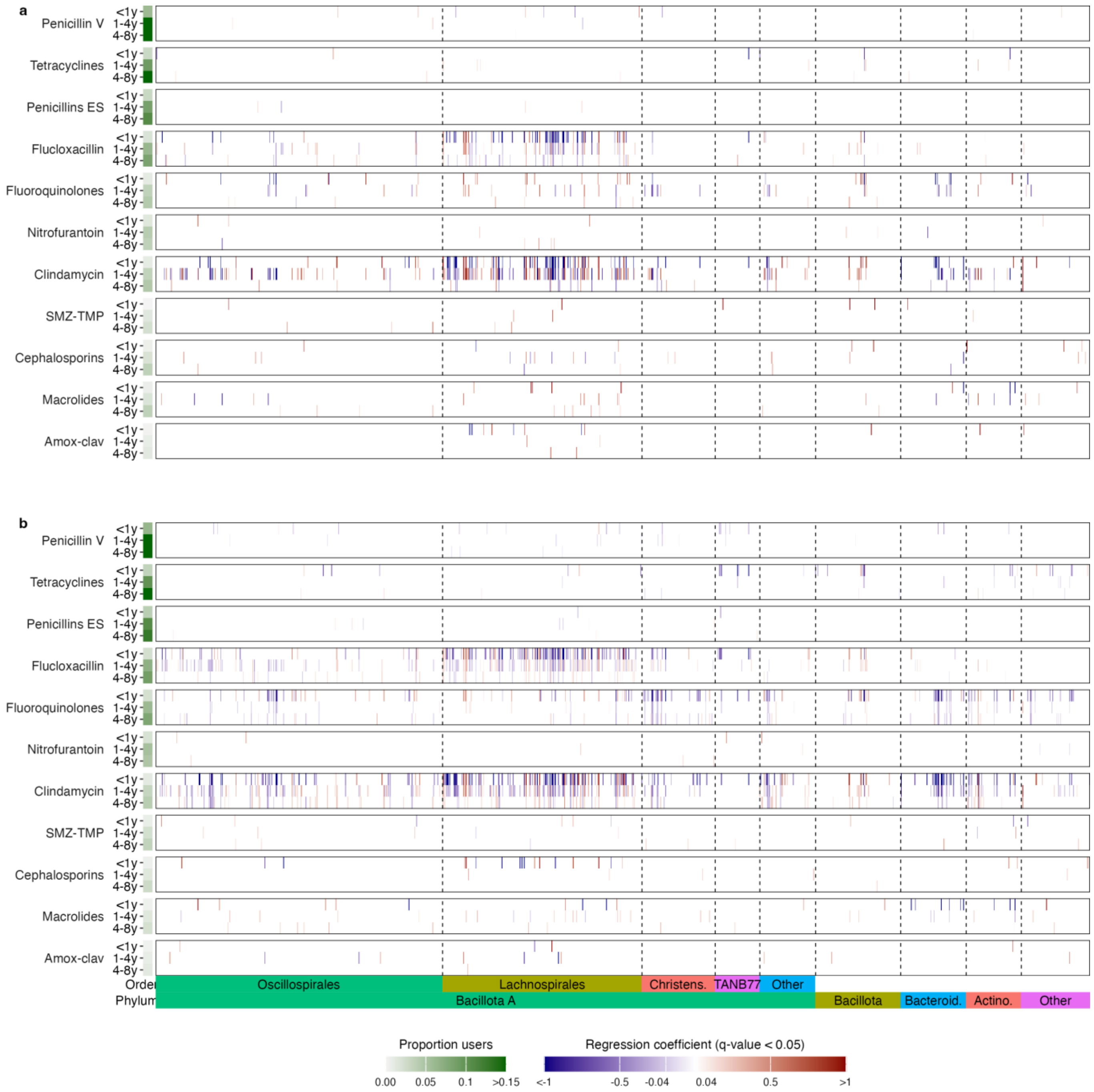

Supplementary Figure 12. Antibiotic use and its associations with abundance of gut microbiome species stratified by age groups. a. ≤ 55 years (n = 4,259), **b**. >55 years (n = 10,715). The associations between the number of antibiotic courses before fecal sampling and species abundance were investigated using regression models adjusted for full-model covariates in three cohorts (SCAPIS, MOS, and SIMPLER, total n = 14,974), followed by meta-analyses. Blue or red vertical bar represents a negative or positive association, respectively. Only significant associations are shown (FDR <0.05). The y-axis displays the 11 antibiotic classes and the periods of the antibiotic courses: <1 year, 1–4 years, and 4–8 years before fecal sampling. The proportion of users indicates the proportion of individuals aged (**a**) ≤ 55 years and (**b**) >55 years who had at least one course of the respective antibiotic in that period. The 1340 species in the x-axis were ordered based on their taxonomy. Phyla are identified at the bottom of the plot. For the phylum *Bacillota* A, the taxonomic orders are also displayed. Penicillins ES: extended-spectrum penicillins; SMZ-TMP: sulphamethoxazole-trimethoprim; Amox-clav: amoxicillin-clavulanic acid; Bacteroid.: *Bacteroidota*; Actino.: *Actinomycetota*; Christens: *Christensenellales.*
