# Supplementary material for: Antibiotic use and gut microbiome composition links from individual-level prescription data of 14,979 individuals
Source: Nat Med. 2026 Mar 11;32(4):1351–61. doi: 10.1038/s41591-026-04284-y (PMC13099378; doi:10.1038/s41591-026-04284-y)
Supplement: Supplementary file 2 — Reporting Summary [file 41591_2026_4284_MOESM2_ESM.pdf]

nature portfolio

Corresponding author(s): Tove Fall

Last updated by author(s): Jan 27, 2026

Reporting Summary

Nature Portfolio wishes to improve the reproducibility of the work that we publish. This form provides structure for consistency and transparency in reporting. For further information on Nature Portfolio policies, see our Editorial Policies and the Editorial Policy Checklist.

Statistics

For all statistical analyses, confirm that the following items are present in the figure legend, table legend, main text, or Methods section.

n/a	Confirmed	
☐	☒	The exact sample size (*n*) for each experimental group/condition, given as a discrete number and unit of measurement
☐	☒	A statement on whether measurements were taken from distinct samples or whether the same sample was measured repeatedly
☐	☒	The statistical test(s) used AND whether they are one- or two-sided<br>*Only common tests should be described solely by name; describe more complex techniques in the Methods section.*
☐	☒	A description of all covariates tested
☐	☒	A description of any assumptions or corrections, such as tests of normality and adjustment for multiple comparisons
☐	☒	A full description of the statistical parameters including central tendency (e.g. means) or other basic estimates (e.g. regression coefficient) AND variation (e.g. standard deviation) or associated estimates of uncertainty (e.g. confidence intervals)
☐	☒	For null hypothesis testing, the test statistic (e.g. *F*, *t*, *r*) with confidence intervals, effect sizes, degrees of freedom and *P* value noted<br>*Give P values as exact values whenever suitable.*
☒	☐	For Bayesian analysis, information on the choice of priors and Markov chain Monte Carlo settings
☒	☐	For hierarchical and complex designs, identification of the appropriate level for tests and full reporting of outcomes
☐	☒	Estimates of effect sizes (e.g. Cohen's *d*, Pearson's *r*), indicating how they were calculated

Our web collection on statistics for biologists contains articles on many of the points above.

Software and code

Policy information about availability of computer code

Data collection	For the collection of the metagenomic sequencing data, the softwares Bowtie 2 v2.4.2 and BWA mem v0.7.17 were used.
Data analysis	All statistical analyses were performed using R version 4.3.2. The R The code used is available at https://github.com/MolEpicUU/antibiotic_gut. R packages used for statistical analyses: lmerTest v.3.1.3, metafor v.4.4.0, emmeans v.1.8.8, car v.3.1.2, ppcor v.1.1, fda v.1.1, and fda.usc v.2.1.0

For manuscripts utilizing custom algorithms or software that are central to the research but not yet described in published literature, software must be made available to editors and reviewers. We strongly encourage code deposition in a community repository (e.g. GitHub). See the Nature Portfolio guidelines for submitting code & software for further information.

Data

Policy information about availability of data

All manuscripts must include a data availability statement. This statement should provide the following information, where applicable:

- Accession codes, unique identifiers, or web links for publicly available datasets
- A description of any restrictions on data availability
- For clinical datasets or third party data, please ensure that the statement adheres to our policy

The data supporting the conclusions of this article were provided by the SCAPIS, SIMPLER, and MOS data offices and contain sensitive personal information protected under privacy laws; therefore, they are not publicly available. Requests for access to data to verify the analyses and findings of this study should be

directed to the corresponding author. An initial response to the request will be provided within two weeks. Data will be shared once a data-sharing agreement has been signed between Uppsala University and the requestor's institution, and following approval from the Swedish Ethical Review Authority (https://etikprovningsmyndigheten.se), and the boards of SCAPIS, SIMPLER, and MOS. Requests for data access for additional research purposes should be directed to the respective cohorts: SCAPIS (https://www.scapis.org/data-access/), SIMPLER (https://www.simpler4health.se/w/sh/en/researchers/data-access), and MOS (https://www.malmo-cohorts.lu.se/application-data-and-samples/applying-samples-mdc-and-mpp). De-hosted anonymized metagenomic sequencing data from SCAPIS are available in the European Nucleotide Archive under accession number PRJEB51353 (https://www.ebi.ac.uk/ena/browser/view/prjeb51353).

Research involving human participants, their data, or biological material

Policy information about studies with human participants or human data. See also policy information about sex, gender (identity/presentation), and sexual orientation and race, ethnicity and racism.

Reporting on sex and gender	Gender -specific analysis were not performed in this study. Sex-stratified analyses were performed for men and women. The interaction between sex and antibiotic use was also investigated. Findings from this study are applicable to general population.<br><br>SCAPIS and MOS: sex was obtained from the Swedish population register.<br>SIMPLER: Invitation sent to all women for mammography screening (identified from the Swedish population register); men were identified from the Swedish population register.
Reporting on race, ethnicity, or other socially relevant groupings	Information on race or ethnicity is not available from the Swedish population-based cohorts. "Country of birth" was used as a covariate in the statistical model and is reported in Table 1.
Population characteristics	SCAPIS enrolled 30,154 women and men aged 50–65 invited from a random sample of residents in areas adjacent to 6 academic hospitals in Sweden between 2013 and 201825.<br><br>SIMPLER encompasses two large population-based studies: the Cohort of Swedish Men (COSM) and the Swedish Mammography Cohort (SMC). The COSM initially enrolled 48,850 men born between 1918 and 1952 living in Västmanland and Örebro counties in 1997. The SMC(n=66,651) invited all women born between 1914 and 1948 living in Uppsala and Västmanland counties between 1987 and 1990. The current study includes a randomly selected subsample from COSM and SMC who were invited for clinical examination for whom gut microbiome data are available.<br><br>MOS includes the adult children and grandchildren of the population-based Malmö Diet and Cancer-Cardiovascular Cohort participants. The recruitment occurred between 2013 and 2021.<br><br>Covariates included in the study were age, sex, smoking status, education level, country of birth, BMI, doctor-diagnosis of inflammatory bowel disease, and chronic pulmonary disease (chronic obstructive pulmonary disease, chronic bronquitis, and emphysema), Charlson Comorbidity Index, and medication use of proton-pump inhibitors, metformin, statins, selective serotonin inhibitors, beta blockers, and antipsychotics. Detailed information is provided on Table 1.
Recruitment	SCAPIS used population registers to randomly invite individuals with age 50-64 living in the areas of Uppsala and Malmö in Sweden. Recruitment ensured that a approximately equal number of men and women were enrolled. MOS invited adult children and grandchildren of participants of the earlier Malmö Cancer Study.<br>SIMPLER: From March 1987 to December 1990, all women living in Uppsala County in Sweden and who were born between 1914 and 1948 and all women living in the Västmanland County born between 1917 and 1948 received an invitation to participate in a population-based mammography screening program. In 1997, all men born between 1918 and 1952 living in Västmanland and Örebro counties in Sweden received an invitation to participate in the study.
Ethics oversight	Ethical approval was obtained from the Swedish Ethical Review Authority (DNR 2018-315 B and amendments 2020-06597 and 2022-06460-02, DNR 2012-594 and amendments 2017-768 and 2020-05611, DNR 2022-06137-01 and amendment DNR 2023-04785-02). All participants in each of the three studies provided written informed consent.

Note that full information on the approval of the study protocol must also be provided in the manuscript.

Field-specific reporting

Please select the one below that is the best fit for your research. If you are not sure, read the appropriate sections before making your selection.

- [x] Life sciences
- [ ] Behavioural & social sciences
- [ ] Ecological, evolutionary & environmental sciences

For a reference copy of the document with all sections, see nature.com/documents/nr-reporting-summary-flat.pdf

Life sciences study design

All studies must disclose on these points even when the disclosure is negative.

Sample size	Sample sizes were determined based on data availability within the included cohorts and predefined inclusion criteria. No formal a priori statistical power calculations were performed. 9816 participants from SCAPIS, 5889 from SIMPLER and 2223 from MOS had fecal metagenomics data available that passed quality control and had consented to have their data linked to population registers (full cover of the Swedish population). After exclusion (see below), 8488 participants in SCAPIS, 4784 in SIMPLER, and 1707 in MOS had full information on the

covariates used in the basic linear regression model. 8488 participants in SCAPIS, 4779 in SIMPLER, and 1707 in MOS had full information on the covariates used in the full linear regression model.

Data exclusions

Participants were excluded if they did not consent to have their data linked to population-registers. Participants with a site visit before July 1, 2013, were excluded as their history of antibiotic use in the past 8 years was not available. To exclude samples collected during antibiotic treatment, we excluded individuals with an antibiotic prescription in the 30 days preceding the study site visit, and participants with prescriptions patterns of antibiotic use for urinary tract infection prophylaxis or acne/rosacea treatment at the time of fecal sampling. Long-term medications are typically dispensed for three-month periods. Therefore, we excluded all participants with a dispensed methenamine prescription in the three months before the fecal sampling. Likewise, we excluded participants with dispensed nitrofurantoin or trimethoprim prescriptions in the 12 weeks before fecal sampling summing to at least 22.5 defined daily doses (DDD), which is equivalent to 50 mg and 100 mg, respectively, once a day for 12 weeks. To exclude long-term users of doxycycline for rosacea, we excluded all participants with a prescription of 40mg of doxycycline tablets in the 12 weeks before the fecal sampling, all participants with one or more prescriptions of 100mg of doxycycline tablets adding up to at least 84 DDD in the last 12 weeks (equivalent to 100mg/day for 12 weeks), at least 56 DDD in the last 8 weeks, or at least 42 DDD in last 6 weeks. To exclude long-term users of tetracycline or lymecycline for rosacea, we excluded all participants with dispensed prescriptions adding up to at least 42 DDD in the last 12 weeks (equivalent to 500mg/day or 300mg/day, respectively, for 12 weeks), at least 28 DDD in the last 8 weeks, or at least 21 DDD in the last 6 weeks.
Because the visit dates were available but not the exact date of fecal sampling, we excluded SCAPIS and MOS participants who a) had an antibiotic prescription between the two visits, b) provided fecal samples >7 days after the second visit, or c) had an interval of >60 days between visits given the uncertainty about sample collection date. Additionally, we excluded those with a diagnosis of chronic pulmonary disease (i.e., chronic pulmonary obstructive disease, chronic bronchitis, and emphysema), and/or inflammatory bowel disease (ulcerative colitis or Crohn's disease), as these conditions often entail a recurrent need for antibiotics and have been associated with substantial alterations in the gut microbiota

In the analysis between species and cardiometabolic biomarkers in SCAPIS, the 743 individuals with diabetes were removed.

Replication

We chose to perform meta-analyses of the statistics from the three population-based rather than a discovery-replication approach. However, the results for each cohort separately are available in the supplementary tables. We observe that associations are consistent in the three cohorts.

Randomization

No randomization was performed in this observational study.

Blinding

No intervention or blinding was performed in this observational study.

Reporting for specific materials, systems and methods

We require information from authors about some types of materials, experimental systems and methods used in many studies. Here, indicate whether each material, system or method listed is relevant to your study. If you are not sure if a list item applies to your research, read the appropriate section before selecting a response.

Materials & experimental systems

n/a	Involved in the study
☒	☐ Antibodies
☒	☐ Eukaryotic cell lines
☒	☐ Palaeontology and archaeology
☒	☐ Animals and other organisms
☐	☐ Clinical data
☒	☐ Dual use research of concern
☒	☐ Plants

Methods

n/a	Involved in the study
☒	☐ ChIP-seq
☒	☐ Flow cytometry
☒	☐ MRI-based neuroimaging

Clinical data

Policy information about clinical studies
All manuscripts should comply with the ICMJE guidelines for publication of clinical research and a completed CONSORT checklist must be included with all submissions.

Clinical trial registration

No clinical trial registration was done for this observational study. Ethical permission was however obtained from the Swedish Ethical Authority for this study.

Study protocol

This is an observational study and no intervention protocol is available. An analysis plan was written before starting the data analysis.

Data collection

SCAPIS participants were recruited between 2013 and 2018. MOS participants were recruited and clinical data obtained between 2013 and 2021. Data from SIMPLER used in this study were obtained in study site visits or questionnaires filled at home between 2012 and 2018

Outcomes

The outcome of this study is gut microbiome composition analyzed using fecal shotgun metagenomics.

Plants

Seed stocks	NA
Novel plant genotypes	NA
Authentication	NA
